# Shedding Light on Graphene Quantum Dots: Key Synthetic Strategies, Characterization Tools, and Cutting-Edge Applications

**DOI:** 10.3390/ma14206153

**Published:** 2021-10-17

**Authors:** Slađana Dorontić, Svetlana Jovanović, Aurelio Bonasera

**Affiliations:** 1 “Vinča” Institute of Nuclear Sciences—National Institute of the Republic of Serbia, University of Belgrade, P.O. Box 522, 11000 Belgrade, Serbia; sladjanaseatovic93@gmail.com; 2 Palermo Research Unit, Department of Physics and Chemistry—Emilio Segrè, University of Palermo, 90128 Palermo, Italy

**Keywords:** dye-sensitized solar cells, energy storage, graphene quantum dots, hybrid materials, synthesis

## Abstract

During the last 20 years, the scientific community has shown growing interest towards carbonaceous nanomaterials due to their appealing mechanical, thermal, and optical features, depending on the specific nanoforms. Among these, graphene quantum dots (GQDs) recently emerged as one of the most promising nanomaterials due to their outstanding electrical properties, chemical stability, and intense and tunable photoluminescence, as it is witnessed by a booming number of reported applications, ranging from the biological field to the photovoltaic market. To date, a plethora of synthetic protocols have been investigated to modulate the portfolio of features that GQDs possess and to facilitate the use of these materials for target applications. Considering the number of publications and the rapid evolution of this flourishing field of research, this review aims at providing a broad overview of the most widely established synthetic protocols and offering a detailed review of some specific applications that are attracting researchers’ interest.

## 1. Graphene Quantum Dots: Definition, Structure, and Properties

The first quarter of the XXI century can be envisaged as a pivotal moment in the field of materials research, due to the high number of innovative materials introduced with the declared purpose of minimizing the demand for rare, precious elements, or introducing new applications and new fields of research [1,2,3,4]. In this view, organic nanomaterials drew the attention of the scientific community, and one of the most evident results is represented by the rapid evolution of carbonaceous nanomaterials (CNMs) [5,6]. This family of materials was initially constituted by fullerenes [7] and carbon nanotubes [8], the first isolated forms, but, later, nanoonions, nanohorns, nanodiamonds, and graphene were discovered, and an increasing number of research teams focused on the implementation and/or modulation of their extremely appealing electrical and mechanical properties. As a general feature, these materials showed, in principle, limited solubility in the most common media, limiting their application or processability. Considering carbon nanotubes and graphene, functionalization protocols can be helpful, but the payback consists of the modification of their properties or their loss [9,10]. Graphene quantum dots (GQDs), one of the most recently discovered CNMs, are currently the focus of the scientific community because, besides their appealing electrical and optical properties, they are typically characterized by high solubility, thus making their processing simple and cheap.

GQDs are zero-dimensional (0D) carbon-based nanomaterials, with graphene sheets as key structural features, with an overall size lower than 10 nm and a thickness below 2 nm [11,12]. Graphene is a monoatomic layer of sp^2^ hybridized C-atoms organized as a succession of fused benzene rings, with a π-electron cloud below and above the graphene plane, which is responsible for the elevated electrical conductivity that is typical of this CNM. An infinite exciton Bohr radius is characteristic of graphene [13], while GQDs are a 0D material that can be conceptually described as the result of 2D graphene size reduction.

Besides graphene, GQDs have oxygen-containing functional groups, where O-atoms are involved in the formation of carboxyl, carbonyl, epoxy, and ethoxy moieties [14]. Due to this structure, GQDs possess properties inherited from 2D graphene, while their small size allows them to act as quantum dots [15,16]. Thus, they show astonishing optical and physical characteristics, such as non-zero bandgap, edge, and quantum confinement effects.

**Figure 1:**
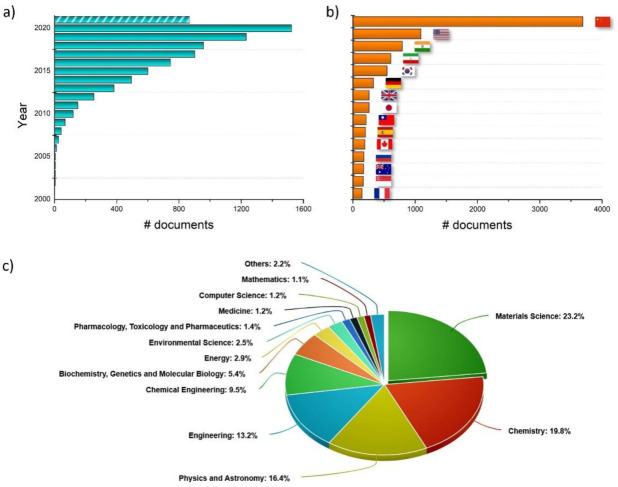
Pictorial representation of the data retrieved by Scopus database: (**a**) number of scientific reports per year, (**b**) distribution of the GQD literature with respect to the researchers’ geographic localization, (**c**) distribution of the GQD literature with respect to the research field.

GQDs started to attracted the attention of the community recently. Their existence was already postulated at the beginning of 2000s, but the first evidence came in 2004 with the report by Xu et al., who firstly identified these materials as the degradation product of single-walled carbon nanotubes [17]. Interest quickly rose around these tiny but brilliant aromatic islands, and the cornerstone work by Ponomarenko and Geim in 2008 [18] introduced new scenarios, as there was an established and reproducible route to designing and isolating GQDs. Since then, the scientific interest in GQDs continued to grow, as new strategies for their synthesis were investigated and new applications pursued. As the Scopus literature database reports (see Figure 1), in the last 5 years and up to September 2021, close to 5500 scientific reports were published, thus supporting this fertile field of investigation. Data also confirm that the principal features triggering the attention of chemists, physicists, and engineers are their optical, mechanical, and thermal characteristics, as most publications are related to materials science, chemistry, and engineering, while biological and medical applications are not explored to the same extent.

One of the most interesting and appealing properties of GQDs is their photoluminescence. It was reported in 2010 by Pan et al. [19], and they attributed it to the quantum confinement effect. Shortly after, scientists tried to tune the properties of such interesting organic scaffolds and, two years later, GQDs doped with a heteroatom (nitrogen) were successfully produced [20]. These studies have increased the interest in GQDs, rendering them a key member of the CNM family. Thanks to their water solubility, biocompatibility, small size, economically favorable synthesis, high reaction yields, simple production, stabile and long photoluminescence, and resistivity to photobleaching, they have placed conventional organic fluorophores and semiconducting dots in the shadows. Apart from bioimaging, GQDs are currently being investigated as an agent in the photodynamic therapy of carcinoma and bacteria, in sensors and photocatalysis, and as components of filtration systems [21,22] and conductive inks [23], but there is rising interest in energy production and energy storage applications [24]. The present energetic crisis is calling for urgent action devoted to the fabrication of innovative devices, able either to efficiently convert solar light into electricity or store it with minor losses if electrical power is produced when the grid is not able (or does not need) to generate further energy. In such deeply investigated fields of research, GQDs represent an opportunity to be exploited, due to their modulable properties, easy processability, and cheap production costs. Based on this background, the aim of the review is to offer the readership a comprehensive revision of the literature focusing on GQD synthesis, which is the most exploited tool for modulating their electronic and optical profiles, with a focus on the latest findings (Section 2); the most common characterization techniques will be introduced as well, to help those readers who are not familiar with GQD research (Section 3). This knowledge will be the necessary premise to discuss GQDs’ applications in the energy production and energy storage fields in the second half of the article (Section 4); here, we spotlight how GQDs can be applied to these technologies as photosensitizers or as the main constituents of other important parts of cutting-edge devices. Before moving to the core of the discussion, some updated review articles are here acknowledged; these detail other specific application fields that are not the main topic of this review but could arouse the interest of some readers looking for insights into electrochemical sensor applications [25], photonics [26], water treatment technology, and catalysis [27].

## 2. Graphene Quantum Dots Synthetic Strategies

Among several properties, the electronic properties of GQDs and the resulting optical features are of great interest, and their modulation is attracting scientists’ attention.

The optical properties of GQDs depend on the electronic structure and bandgap energy, edge configuration, size of dots, presence of dopants, doping method used, as well as the resulting structure of the GQDs [28]. Computational models are powerful tools to guide laboratory activities [29]; models of armchair (AM) and zigzag (ZZ) edge group conformations showed that the bandgap varies from 3.27 eV in AM to 0.27 eV in ZZ [30]. When the diameter of GQDs increases, the energy bandgap and the exciton binding energy decrease, and their values tend to be lower for ZZ conformation models. However, current synthetic approaches do not always guarantee the production of a sole, specific GQD product, but a mixture of dots with a certain polydispersion of the nanomaterials in terms of structural parameters and composition. For this reason, experimental investigation is privileged at the expense of computational models; researchers are rarely working to improve synthetic strategies, to achieve the design of protocols able to return monodispersed structures and more predictable properties. Besides this, optimization in terms of yields and the necessity to target low-cost and environmentally sustainable protocols are piloting laboratory activities as well [31].

**Figure 2:**
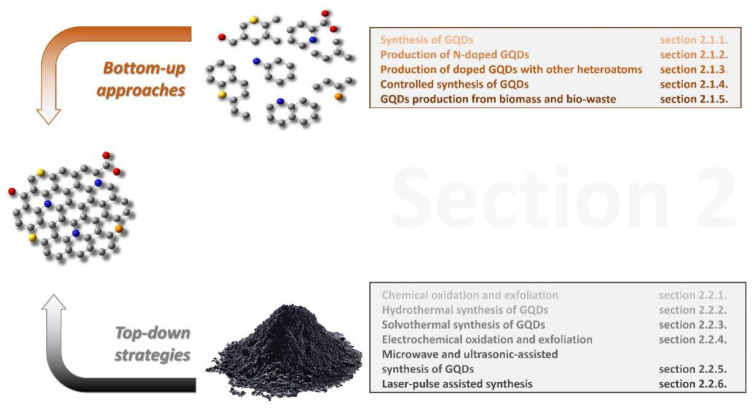
The synthetic designs reviewed in the following subsections. In the end, laboratory equipment availability or specific desired features will drive the interest of the readers towards bottom-up or top-down strategies.

Looking at the broad scenario, all approaches to GQD synthesis can be grouped into two main categories: bottom-up and top-down strategies. The starting material in the bottom-up synthesis of GQDs is low-molecular-mass organic molecules, which are exposed to different treatments to induce their graphitization. A top-down approach is based on the disruption of larger graphene-based materials such as graphite, graphene, carbon nanotubes, carbon black, or fullerenes, into small, 0D dots. The tools to assist scientists in breaking these large 1D, 2D, or 3D materials can be either chemical oxidative agents, microwave irradiation, ultrasound, hydrothermal conditions, laser irradiation, electrical currents, or plasma [32,33,34]. In the following subsection, both types of methods as well as the properties of produced dots will be discussed in detail, starting with bottom-up strategies (Section 2.1) and later moving on to top-down approaches (Section 2.2), as pictorially summarized in Figure 2. A more detailed index will be provided at the beginning of each specific section.

### 2.1. GQDs Bottom-Up Strategies

Focusing on bottom-up approaches, GQDs are produced using small organic molecules as a pristine material, which either undergoes physical treatments such as thermal decomposition, microwave irradiation, etc., or is involved in an organic chemical reaction. The following discussion will detail generic synthetic routes (Section 2.1.1), then shifting the focus to N-doped GQDs (Section 2.1.2) and dots integrating different heteroatoms (Section 2.1.3). Some insight into “controlled synthesis” is provided in Section 2.1.4. Recently, environmental concerns and the economic valorization of less valuable materials resulted in taking into consideration innovative routes based on the implementation of biomass and biowaste as starting materials (see Section 2.1.5).

#### 2.1.1. Synthesis of GQDs

The carbonization of small molecules by heating at a temperature above their melting point is one of the simplest synthetic designs; the heating process induces nucleation phenomena, followed by condensation and the production of GQDs [14]. Carbonization and aromatization processes can be induced by a microwave [35], and it is an appealing alternative considering that this method is simple, fast, and it does not require expensive laboratory equipment. Staring materials for synthesis can be different precursors that contain only C, O, and H in their molecular scaffold: glucose [36,37], citric acid [38,39], humic acid [40], and solvents such as acetylacetone [35], etc., are among the most popular choices due to their ubiquitous presence in chemistry laboratories.

The thermochemical decomposition or pyrolysis of citric acid (CA) is a frequently used approach for GQD production. GQDs are efficiently produced at a relatively low temperature (200 °C), in an ambient atmosphere, and within short times (40 min) [41]. It is observed that the temperature of annealing has an impact on the photoluminescence properties of GQDs. The average size of these dots was reported to be 4 nm, the interlayer distance 0.24 nm; the ratio between two bands in the Raman spectrum of GQDs (D- and G-bands), so-called I_D_/I_G_, was 1 (a more detailed discussion of Raman spectra interpretation is provided in Section 3.2), and the photoluminescence (PL) emission band was centered at 450 nm. With the increase in annealing temperature from room temperature to 300 °C, the PL intensity maximum was redshifted from 500 to 545 nm. Moreover, in samples annealed at different temperatures (100, 200, and 300 °C), PL was gradually quenched. This method is very simple, fast, and environmentally friendly. Pyrolysis of trisodium citrate directly by heated at 200 °C for 4 min leads to GQD formation [42]. An average diameter is reported to be 1.3 nm, with a height of 0.6 nm, while lattice spacing of 0.22 nm is assigned to (1120) lattice fringes of graphite. A bright blue PL emission is detected, while the PL quantum yield (QY) is measured to be 3.6%. In the structure of GQDs, Na atoms were detected, apart from C, O, and H, which come from the precursor salt. GQDs show high PL stability under different conditions (good salt tolerance, stability in pH range 5.5 ÷ 11.0, unchanging PL intensity at 90 °C). The PL of GQDs remained stable for more than 3 months at room temperature. Blue GQDs are also produced by the thermal decomposition of CA using a furnace or a domestic or scientific microwave synthesizer [43]. Hydrophobic GQDs were produced by the thermal decomposition of CA in the presence of octadecyl amine at 200 °C for 14 min, at atmospheric pressure, and resulted in blue emission at 460 nm, at excitation of 360 nm [44]. One more procedure for the synthesis of GQDs from CA consists of its dispersion in ethanol and octadecyl amine, filtering the precipitate out of the solution, heating the mixture at 65 °C for 24 h, then adding glycine and heating the solution at 200 °C for 3 h [45,46]. The so-produced dots emit blue light when they are excited with UV light.

More recently, SiC was used for the production of high-quality GQDs [47]. Lee and coworkers described a procedure wherein SiC plates were heated in a vacuum furnace under an Ar:H_2_ atmosphere (96:4) at 1500 °C for 30 min. GQDs were detached from SiC plates by gentle sonication. Produced dots were 2.4 nm in diameter, with lattice spacing of 0.25 nm, and PL QY was 30.9% at excitation of 300 nm. This procedure demands the use of a high-temperature furnace, special safety equipment to work with H_2_, and must be performed by specialized personnel trained for work with H_2_. Thus, the use of an explosive gas is the main disadvantage of this procedure. One more concern comes from the high price of SiC.

Phenol is another common organic compound used as a starting material in GQD synthesis [48]. In the procedure reported by Liu et al., after dispersing phenol in acetone, hydrogen peroxide was added and the mixture was heated in an autoclave at 200 °C for 20 h and exposed to an external magnetic field with an intensity of 9 T. Produced dots were 3.6 nm in diameter, PL QY was 16.67%, and they showed strong absorption in the NIR-II region at 1070 nm. A high graphitization degree was detected by Raman spectroscopy, due to the high value of the intensity ratio between the G and D bands (1.21). The interplanar distance was 0.346 nm and assigned to the (002) planes of the graphite lattice. Only C- and O-atomic species were detected in the structure of these dots. This procedure demands the use of an autoclave for 20 h as well as an external magnetic field of 9T. Prolonged heating and the application of an external magnetic field increases the complexity of the synthetic procedure, while the starting chemicals are affordable.

In another study, various solvents were used for the solvothermal production of GQDs: ethylene glycol (EG), dimethylformamide (DMF), acetone (AC), toluene (T), glycerol (G), ethylenediamine (EDA), methanol, ethanol, and carbon tetrachloride [49]. Researchers discovered that organic solvents undergo decomposition at a temperature of 220 °C or lower, producing GQDs without the use of any catalyst. EG, DMF, AC, T, G, and EDA produced dots with PL QY from 3.73 to 19.02% and a diameter of ca. 2 nm. When acetylacetone (AcAc) was used by Umrao and coworkers as a starting material, they mixed 50 mL of AcAc with two drops of deionized water (DI) in a quartz bowl, and exposed it to microwave irradiation with a temperature limit of 200 °C and power of 800 W for 5 min [35]. The proposed procedure is very fast, simple, and requires only the use of an MW reactor.

Polyacrylonitrile (PAN) nanofibers were also used as a starting material for GQD production [50]. Dispersion of PAN in DMF was deposited by electrospinning on a collecting metal plate. Then, the material was heated to achieve carbonization/graphitization at 1500 °C for 5 h under a nitrogen atmosphere. In the next oxidative step, the crude material was heated after dissolution in a mixture of H_2_SO_4_ and HNO_3_ for 1 day. The average diameter of dots was 10.98 nm, interlayer spacing was 0.19 nm, the optical bandgap from the Tauc plot was 3.4 eV and 3.9 eV, and the dots emitted blue light.

#### 2.1.2. Production of N-Doped GQDs

To tune the electronic and optical features of GQDs, a simple strategy consists of doping the dots with heteroatoms, nitrogen in particular; the reason is due to the similar atomic radius (0.70 Å) that N-atoms have compared to carbon ones (0.77 Å), and their higher electronegativity (χ_N_ = 3.04) than C (χ_C_ = 2.55). This makes nitrogen incorporation into the carbon network achievable. Moreover, it has been demonstrated that edge-N-doping modulates the energy level of excited states and increases the radiation transition probability, leading to the improvement of PL properties. N-doping reduces the energy difference between low-lying excited states and increases the radiative transition [51]. Due to the difference in electronegativity, N-doped GQDs (N-GQDs) are usually selected for application in electronics, energy storage, and catalysis [52]. For this reason, the following section will focus on N-GQDs, due to the large research interest in their production; the discussion of GQDs containing other heteroatoms will be offered in Section 2.1.3.

N-GQDs can be easily produced starting from 1,3,6-trinitropyrene, heating the material in an autoclave at 200 °C for 10 h [53], or using CA and EDA, with the latter chemical acting as a N-donating species [54]. Urea (U) and CA were used as precursors in the production of white GQDs [55]. DMF was used as a solvent, and the reaction was conducted at 180 °C for 0.5 to 8 h. Produced dots were 3 ÷ 6 nm in diameter, with the interlayer spacing of 0.24 nm, and C- (79.40 at.%), N- (7.64 at.%), and O-atoms (12.96 at.%) were detected in the samples, while the I_D_/I_G_ ratio was 1.42. Dots emitted different colors, from blue and white to green, depending on their size.

One more protocol for obtaining N-GQDs is based on heating a mixture of CA and EDA in water, at 70 °C for 30 h [56]. Dots’ size varied between 3 and 5 nm, the lattice spacing was 0.31 nm and assigned to the (100) spacing of the graphitic lattice, and the emission peaks were from 466 to 529 nm at excitation wavelengths ranging from 310 to 440 nm, respectively. All these methods included the application of a hydrothermal reaction and heating up to 200 °C. Synthesis includes only one step (heating for up to 10 h) and it is followed by filtration and dialysis. Due to the low price of chemical and synthetic approaches, these techniques are transferable to large scales, are economically acceptable, and they are not time-consuming

There are also a few approaches for the production of N-doped GQDs in a much shorter time, at atmospheric pressure. In only 20 min, without water or solvents, N-GQDs were produced by heating CA and histidine, which served as a surface passivation agent at 160 °C, at atmospheric pressure [57]. Green emitting dots were produced (emission peak 470 nm), with a size around 2 nm, lattice spacing of 0.212 nm assigned to the (100) graphene planes, and PL QY of 62.8%. Dots were rich in both carboxyl groups and imidazole fragments. According to this method, precursors were simply heated on a hot plate, and dots were then cleaned using only dialysis. This method seems the simplest, with no demand for special equipment and with affordable chemicals. However, the authors did not report the reaction yield.

N-GQDs were synthesized from uric acid, by heating the precursor in concentrated sulfuric acid at 200 °C for 1 h [58], or d-glucose in the presence of 1-hexadecylamine at a temperature of only 60 °C, where single crystalline dots were detected [59]. N-GQDs have been isolated by hydrothermal treatment at 150 °C for 4 h with a glucose aqueous solution mixed with ammonia and H_2_O_2_ [60]. Produced dots were 4.5 nm in diameter, with a d-spacing of 0.24 nm assigned to the (1120) lattice fringes of graphene, with 63.1 at.% of carbon atoms, 30.1 at.% of oxygen ones, and 6.8 at.% of nitrogen atoms, where N was present only as part of the amino groups. PL QY was 32.8% and the emission peak was localized at 500 nm. GQDs with only amino functional groups were also produced by heating L-cysteine at 215 °C in an oil bath at atmospheric pressure, for 30 min [61]. Produced dots were between 4 and 8 nm, with interplanar spacing around 0.235 nm; the Raman ratio I_D_/I_G_ was 0.82, and the highest-intensity emission peak at 425 nm was detected for an excitation wavelength of 360 nm.

N-GQDs were obtained by the simple heating of a water solution of ammonium citrate at 200 °C under reflux, at atmospheric pressure [62]; the produced dots were 1–5 nm in lateral size, around 3 nm in height, with the center of the emission band at 433 nm upon excitation at 357 nm.

One-step microwave-assisted pyrolysis of an aspartic acid (Asp) and NH_4_HCO_3_ mixture has been successfully investigated by Zhang et al. [63], with an average diameter of 2.1 nm and interlayer distance between 0.25 and 0.33 nm, 14% PL QY, and PL emission at 440 nm upon excitation at 350 nm.

CA hydrothermal treatment at 160 °C for 4 h was also exploited for the preparation of N-GQDs [64]. In this case, dots were functionalized by heating at 700 °C for 1 h in N_2_ gas. Then, KOH and carbonized GQD were mixed at the weight ratio of 2:1 and heated at 800 °C for 1 h under an inert gas atmosphere. Produced dots were below 3 nm, with a height of 4.8 nm, and the emission peak was at 450 nm when dots were excited with 360 nm, with QY PL of 9.8%. Another hydrothermal protocol using dopamine as a starting material produced dots with the size of 3.4 nm, the interlayer distance of 0.24 nm, and high PL QY of 34%, with yellow emission and with high structural disorder (Raman I_D_/I_G_ ratio of 1.78) [65].

*p*-coumaric acid has been investigated as another source of N-GQDs, after its dispersion in anhydrous ethanol, and in the presence of *p*-phenylenediamine as a source of N-atoms [66]. The mixture was heated at 180 °C for 24 h. Dots were 3.8 nm in diameter, the lattice spacing was 0.24 nm, and the Raman spectra showed large disorder (I_D_/I_G_ 1.92), while AFM suggested 1–3 graphene layers in the GQDs’ structure. In the GQDs’ structure, 12.7 at.% of N was measured. Blue emission at 463 nm was detected when dots were excited with 365 nm.

N-GQDs were produced by mixing ammonium fluoroborate (H_4_BF_4_N, 99.5%) and trisodium citrate in water followed by heating in an autoclave at 200 °C for 5 h [67]. Dots that were 21 nm in diameter and 3 ÷ 4 nm height emitted blue light when they were excited with UV light.

Heating of benzimidazole dispersed in absolute ethanol for 6 h at 180 °C resulted in N-GQDs formation [68]. Dots emitted blue light (430 nm) at excitation of 370 nm and showed a very high PL QY of 25%. The average particle size was 3.8 nm, while the lattice spacing around 0.24 nm was assigned to the (100) lattice fringe of graphene. The thickness of the dots was 0.76 nm, meaning that they consisted of 1–2 graphene layers. A very low defect level was observed in the Raman spectra considering that the intensity ratio between the D and G bands was 0.42. N was incorporated into the GQDs’ structure as pyridinic N, pyrrolic N, and amino groups. The highest intensity of the emission peak at 435 nm was detected when dots were excited with 357 nm.

N-GQDs were obtained from chitosan in a bottom-up procedure using a direct-current (DC) Ar–microplasma electrochemical reactor [69]. Obtained dots were 6.39 nm in diameter, with PL QY up to 30%, and the center of the emission band was at 474 nm.

The effect of U and NH_3_ on the structure of GQDs produced by CA decomposition was studied too [70]. CA and NH_3_ were heated in autoclaves for 10 h at 200 °C (A-GQDs), while CA and U were exposed to 180 °C for 4 h (U-GQDs). The higher at.% of N (12.83) was achieved for U-GQDs compared to A-GQDs (10.68). Both types of dots showed high PL QYs, 45.4% and 54% for A- and U-GQDs, respectively. They had different diameters, 16.0 ÷ 20.5 nm and 1.4 ÷ 1.8 nm, for A-GQDs and U-GQDs. Blue emission was detected from A-GQDs and U-GQDs at 442 and 457 nm when they were excited with 365 nm.

N-GQDs can emit near-infrared light [71]. By the solvothermal treatment of an indigo ethanol solution, at 160 °C for 12 h, N-GQDs were produced. The content of N atoms was 10.8 at.% in the form of aromatic and amino groups. The reaction yield was extremely high, close to 99 wt.%. The optical bandgap was 2.3 eV from the Tauc plot. The highest PL emission was detected at 598 nm at excitation of 534 nm. The PL QY was 76% for this excitation. The diameter of these dots was 2.7 nm. When the reaction mixture was heated for 24 h, dots that emitted at 689 nm upon excitation of 602 nm were produced.

In conclusion, N-doped GQDs possess a high PL QY. Through the modulation of the experimental conditions (precursors, solvent, reaction time, C/N source ratio), the size, structure, solubility, and color of emitted light can be tuned. Such a variety of protocols makes GQDs a flexible platform for preparing materials designed for diverse and specific applications. One of the disadvantages can be ascribed to the cleaning stage time demand, necessary to perform efficient dialysis, filtration, and centrifugation steps, but the high size uniformity, hydrophilicity, and optical quality of the obtained dots make these routes promising for further investigation. Dots can be synthesized according to protocols that are based on the use of a broad library of starting materials, with approaches privileging the use of nontoxic, nonhazardous precursors or harmful acids and/or organic solvents. Moreover, several methods do not require the use of specialized laboratory equipment and are conducted at atmospheric pressure, which is an important advantage when looking at the budget designated for laboratory activities. Several methods comply with these key issues [57,59,61]. However, some missing information in the literature does not allow us to provide a more complete survey of the field. Parameters such as process efficiency or reproducibility are often omitted from the discussion, even if their importance is crucial to move such synthetic procedures beyond the laboratory scale and towards industrial application.

#### 2.1.3. Production of Doped GQDs with Other Heteroatoms

S-doped GQDs were produced by Bian et al. from 1,3,6-trinitropyreneas as a source of C-atoms and 3-mercaptopropionic acid (MPA) as the sulfur donor precursor [72]. The hydrothermal reactor was heated at 200 °C for 10 h. Produced dots had an emission band centered at 450 nm and an average diameter of 2.5 nm. Another study detailed by Kadian and coworkers examined the pyrolysis of CA and MPA heated at 200 °C for 10 min under atmospheric pressure [73], resulting in blue-emitting dots (460 nm) upon UV excitation (360 nm). Dots had sulfides as well as sulfone-functional groups; both S-doped dots had similar blue emission, while the lack of reaction yield data does not allow the readers to discern which approach is more efficient.

N,S-doped GQDs were produced by the carbonization of CA with thiourea (TU) at 160 °C for 4 h with QY of 71%, a diameter of 3.1 nm, I_D_/I_G_ 0.77, and 1–5 layers in the structure [74]. In a similar study [75], the heating time was set at 8 h, and the produced dots emitted blue light (462 nm) when excited at 400 nm. Qu et al. modified this synthetic strategy, dispersing CA and TU in DMF and heating the mixture in an autoclave at 180 °C for 8 h, followed by filtration and dialysis [76]. Produced dots had 62.8 at.% of C, 14.3 at.% of N, 8.1 at.% of H, and 9.8 at.% of S; the Raman intensity ratio I_D_/I_G_ was 0.94, the diameter was 4.5 nm, and three excitation-wavelength-independent PL regions were observed, with emission peaks at 440 (blue), 550 (green), and 640 nm (red) at excitation wavelengths from 340 to 420 nm, 460 to 540 nm, and 560 to 620 nm, respectively [76]. This behavior was ascribed to the presence of three different chromophores: the C=O, C=N, and C=S groups in conjugation with the graphene core. S,N-doped GQDs were produced from the polymer poly(3-alkylthiophene) (P3AT) [77]. One more approach for the production of S,N-GQDs from CA and U was assisted by infrared-light irradiation [78]. Samples were produced by the heating of a powdered sample in an infrared furnace at 200 °C, for 40 min. The highest production yield was 43.2 wt.%, with dots characterized by a diameter ranging between 5 and 10 nm, and the lattice distance of ca. 0.22 nm corresponding to the (1120) lattice fringes of graphene sheets. The most intense emission feature was detected at 450 nm, with PL QY up to 22.2%. The polymer was dispersed in NaOH solution, which served as the activating agent. The basic mixture was hydrothermally treated at 170 °C for 20 h, followed by filtration and dialysis. Dots had emission peaks extended to 700 nm and low Raman I_D_/I_G_ values (0.48–0.56). One more S, N-doped GQDs protocol was reported by Shen et al., consisting of the dissolution of 1,3,6-trinitropyrene (TNP) and TU (200 mM) in NaOH (10 mM) containing DMF, and heated at 200 °C for 10 h [79]. These dots were characterized by a diameter of 2.1 nm and intense blue PL with a high QY value (23.2%). S,N-doped GQDs were produced by the hydrothermal treatment of CA and L-cysteine at 180 °C for 6 h [80], characterized by an intense emission peak centered at 421 nm (λ_exc_ = 345 nm) and a diameter between 4 and 10 nm.

Looking at very recent publications, Daugherty and coworkers reported the isolation of S,N-doped GQDs to be applied for redox flow batteries [81]. Here, a solid blend of CA, TU, and (NH_4_)_2_SO_4_ was prepared by means of a three-dimensional mixer with a Zr sphere, treated in an IR furnace with six medium-wave IR heaters (80 kW m^−2^); the thermal pyrolysis process was carried out in air at 280 °C for 30 min. Further separation and washing steps returned dots with a particle size distribution in the range of 4–10 nm, with an average particle size of ~6.2 nm with 41 wt.% yield. The blue color and PL QY as high as 25.5% were consistent with the previously mentioned procedures, but the very high yield makes this protocol very appealing. Other papers report hydrothermal approaches and procedures for the preparation of S,N-doped GQDs [82,83] but, besides the intriguing applications, they mostly rely on previously reported procedures; thus, they do not introduce any progression in the design of new synthetic strategies.

S,P-doped GQDs were obtained by the hydrothermal reaction of CA, sodium phytate (a natural antioxidant occurring in food matrices), and Na_2_SO_4_, at 180 °C for 7 h, followed by centrifugation and dialysis [84]. Herein, a high doping level was achieved: 9.66 at.% for S and 3.34 at.% for P. Dots were 3.5 nm in diameter, with interplanar spacing of 0.28 nm, with a PL QY of 15.7% and blue emission upon UV illumination.

A minor number of reports focus on boron-doping strategies. B-doped GQDs were produced by molecular fusion between 1,3,6-trinitropyrene (carbon source) and borax (dopant) in sodium hydroxide (alkaline medium) under hydrothermal conditions at 200 °C for 6 h, and isolated by dialysis [85]. The reaction yield was 71 wt.%, and it produced dots that were 0.9–1.1 nm thick and 2 nm in diameter, with a lattice parameter of 0.24 nm, which was related to the (100) lattice of graphene, and green-colored emission.

N,B-doped GQDs were produced using a domestic microwave oven, where an aqueous solution of CA, U, and boric acid was heated at 750 W for 5 min under ambient conditions [86]. Produced dots had 12–22 at.% for B-atoms, N in the range of 16–24 at.%, and O 35–45 at.%. PL QY was up to 31%, and the emission band was localized at 450 nm.

Before closing the section, two more unique cases are briefly described: K-doped GQD production has been achieved by the hydrothermal treatment of a sucrose and potassium hydroxide aqueous solution carried out at 170 °C for 4 h [87]; Cl-doped GQDs were produced by the hydrothermal treatment of a solution of fructose and HCl at 170 °C for 4 h [88], resulting in the isolation of dots with a diameter of 5.4 nm and 2 at.% of incorporated Cl.

A wide range of heteroatom precursors allows the synthesis of GQDs with higher PL QY, especially in the case of N,S-doped GQDs, where values of up to 71% were measured. The production of multicolored GQDs (from blue to red) is possible. Moreover, Cl-GQDs showed great optical diversity (emission in the blue, white, orange, green, and red regions of the spectrum). The highest reaction yield was detected for the production of B-doped GQDs from 1,3,6-trinitropyrene and borax, with a value of 71%. This method seems to be most efficient production method. Most of these procedures were performed in hydrothermal conditions in a particular time interval (4–20 h). By applying microwave and infrared irradiation, significant shortening of the reaction time was achieved (5 min in the case of the microwave-assisted approach). In summary, it can be concluded that functionalization with heteroatoms such as N, S, B, and Cl leads to the production of multicolored GQDs with high values for PL QY. Precursors for synthesis of GQDs, conditions, reaction yields, and the most important characterization data are listed in Table 1.

#### 2.1.4. Controlled Synthesis of GQDs

Apart from pyrolysis, there are a few additional methods used for controlled GQD production. In this paper, the term “controlled synthesis” is limited to approaches to GQD production using strictly organic chemical reactions. Here, small organic molecules are used as reactants. These reactions follow a precise stoichiometry, leading to the formation of only one product, GQDs with a number of C-atoms depending on the reaction stoichiometry. Thus, dots produced according to this strategy are uniform in chemical composition, size, and optical properties. These reactions are based on oxidative condensation reactions.

These reactions are based on oxidative condensation reactions. Yan et al. produced colloidal GQDs with well-defined and tunable size by the oxidative condensation of polyphenylene dendritic precursors [13]. As a result, GQDs with 168, 132, and 170 conjugated C-atoms were obtained. A key factor in controlling their size was methanol, which was used to quench oxidative condensation. Based on this research, Wang and collaborators produced fluorescent water-soluble GQDs from pyrene and their derivates composed from four fused carbon rings in a structure similar to graphene cells [93]. This synthesis was based on the nitration of pyrene in nitric acid and condensation under hydrothermal conditions in an alkaline aqueous solution, where alkaline species play an important role in size control, functionalization, and optical properties. As a result, single-crystal, defect-free GQDs were obtained. These GQDs exhibited excitation-independent PL (Section 3.3) due to their crystalline structure and ideal edge state, with a high PL QY (45%).

Another approach is the synthesis of graphene molecules using an organic synthetic route [94]. In the reaction between 1,3,5-triethynylbenzene (TB) and tetraphenylcyclopentadienone (TC) at 160 °C in *o*-xylene overnight, polycyclic aromatic hydrocarbons (PAHs) were produced. After purifying the crude reaction, a dehydrogenation reaction was conducted in dichloromethane (DCM) with iron(III) chloride (FeCl_3_) at room temperature, which resulted in the formation of graphene-like fragments. Then, an oxidation reaction in the presence of nitric acid under reflux was conducted to produce oxidized GQDs and sodium borohydride to produce reduced GQDs. Isolated dots were 2.1 and 1.8 nm in size, with a typical d-spacing for graphite (0.24 nm). The maximum emission wavelength of oxidized GQDs was located at 587 nm, which belongs to the orange–red region, and that for reduced GQDs was 530 nm, in the green–yellow region. PL QYs were 1.4 and 3.4% for oxidized and reduced GQDs, respectively.

Other small organic precursors, such as 3-iodo-4-bromoaniline, can be used as well [13]. Using a stepwise Suzuki coupling reaction, poly(phenylene) was produced and, due to the excess of FeCl_3_ in the DCM/nitromethane mixture, GQDs could be finally isolated.

Another approach consists of the Diels–Alder reaction between 1,3,5-triethynylbenzene and tetraphenylcyclopentadienone, followed by the Scholl reaction, or bromobenzene, which undergoes a Friedel–Crafts reaction with dodecanoyl chloride and obtains an intermediate undergoing Wolf–Kishner reduction [95]. These dots consisted of 96 sp^2^-carbon atoms with a diameter of ∼2 nm. Thanks to the controlled conditions, each dot had six alkyl chains and emission bands centered at 653, 719, and 797 nm.

The most efficient control over GQDs’ morphology, chemical structure, and optical properties is granted by approaches that include condensation reactions of different aromatic molecules in the GQDs’ skeleton. Produced dots are uniform in size, shape, and atomic composition. However, these approaches usually include 7–9 synthetic steps, each demanding the consumption of both time and chemicals, and, in the end, a significant amount of residual reagents and solvents is produced as well. The use of toxic chemicals such as organic solvents and strong acids, as well as the time needed for reactions and product cleaning, cannot be avoided, and these are the main disadvantages of such methods. However, if there is a need for GQDs with uniformly distributed size and stable PL, these could be the methods of choice.

#### 2.1.5. GQD Production from Biomass and Biowaste

More recently, scientific attention was directed towards the production of GQDs from biomass/biowaste precursors using green, easy, and low-cost synthetic processes [96].

Thus, S-doped GQDs were produced from a tropical fruit, durian [89]. The flesh of this fruit was used, introduced in an autoclave in the presence of water and Pt pieces, which served as a catalyst. The material was heated at 150 °C for 12 h, and the reaction yield was 6.8 wt.%. Dots were 4 nm in diameter, 0.5 ÷ 1.0 nm in height, with a lattice parameter of 0.24 nm assigned to the (1120) lattice of graphene. The ratio of I_D_/I_G_ was 0.32, with 3.2 at.% of sulfur within the GQD lattice, extremely high PL QY (79%), and an emission band centered at 601 nm.

Bamboo fibers were also used as a starting material for GQD production [90]. Cellulose nanocrystals were extracted from the biomass and then dispersed in water and heated at 180 °C for 8 h in an autoclave. Produced dots contained only C- and O-atoms, and they possessed a highly ordered lattice (Raman I_D_/I_G_ ratio was 0.72); the diameter of 9.8 nm, the interplanar distance was 0.27 nm, and they showed blue emission upon excitation with 360 nm, with an astonishing PL QY of 38.9%.

Considering cases where biowaste was successfully used for GQD production, Wang and coworkers employed lignin from black liquor [91], a major waste material in the wood industry. Alkali lignin was treated first with *o*-aminobenzenesulfonic acid (*o*-ABSA), and then it was heated in the presence of NaOH in an autoclave at 200 °C for 12 h. Produced dots were 2.2 nm in diameter, with an interlayer distance of 0.21 nm from the (100) graphene plane; the Raman ratio between G and D was 0.98, and the highest PL intensity was detected at 484 nm, when excited with a wavelength of 380 nm, with a calculated PL QY of 20.63%. The bandgap was determined from the Tauc plot, and it was 3.34 eV at the lowest GQD concentration.

GQDs were produced from Miscanthus (MC), a perennial bioenergy crop [92]. After a preliminary purification stage, MC was treated with *p*-toluenesulfonic acid in different concentrations at 80 °C for 20, 60, or 120 min. The obtained dots were highly monodispersed, with a 4.0 nm diameter and 1.8 nm height, while d-spacing was 0.21 nm corresponding to that of (100) planes of graphitic carbon. PL QY was 20.2% and dots emitted blue light. In a different study, MC was used to produce biochar by terminal treatment at 1200 °C and produced both graphene oxide and GQDs [97]. The obtained biochar was mechanochemically cracked in the presence of water or N-methyl pyrrolidone. Produced dots were 6 nm in diameter, with interplanar spacings of 0.22 nm for dots in water and 0.24 nm for those in N-methyl-2-pyrrolidone (NMP).

### 2.2. GQDs Top-Down Synthesis

The top-down synthesis of GQDs implies the oxidative cleavage of various carbon materials, such as graphite [98], carbon nanotubes [99], carbon nanofibers [100], fullerenes [101], graphene oxide (GO) [102], carbon black [103], and even coal [104,105], to obtain quantum dots. The general mechanism is based on the oxidation and exfoliation of bulk carbon materials using different chemical and physical agents, such as strong acids, high temperature and pressure, an electric current, microwave irradiation, ultrasonic waves, a laser beam, and others [106]. Top-down routes can be classified into several groups: chemical oxidation and exfoliation (Section 2.2.1), hydrothermal and solvothermal oxidation (Section 2.2.2 and Section 2.2.3), electrochemical oxidation (Section 2.2.4), microwave-assisted methods and ultrasonic-assisted methods (Section 2.2.5), laser ablation (Section 2.2.6), and others [107], and, according to this classification, they will be separately detailed in the following subsections. At the end of the discussion, Table 2 is provided to summarize the most crucial information concerning the synthetic designs here described.

#### 2.2.1. Chemical Oxidation and Exfoliation

Chemical oxidation includes the oxidation of a carbon-based starting material using strong oxidizing agents such as potassium permanganate, sodium nitrate, H_2_O_2_, or acids, such as sulfur or nitric acids, acting as oxidizers. Oxidizing agents serve as oxidative scissors for cutting bulk material into small-sized quantum dots [108].

Chua et al. synthesized GQDs from C_60_ as a carbon source and 98% sulfuric acid, sodium nitrate, and KMnO_4_ as oxidative agents [101]. The opening of the fullerene cage was achieved by heating at 35 °C for 30 min. The reaction mixture was mixed with DI and heated at 70 °C for a further 15 min. The lateral size of the so-obtained GQDs was in the range of 7–10 nm, and their average height was 0.7 nm. Luminescence was observed in the yellow–red region, with the maximum emission peaks at 630 and 700 nm. Kaciulis et al. used similar experimental conditions (H_2_SO_4_, KMnO_4_, NaNO_3_) [109], obtaining very small GQDs, with lateral sizes between 1 and 2.5 nm.

The chemical oxidation and exfoliation of graphite materials leads to GQD production as well [110,111,112]. Shengnan et al. used multilayer GO as a starting material [112]. A commercial GO was suspended in a mixture that contained sulfuric and nitric acid and heated under reflux for 24 h at a temperature of 110 °C. The average size of the obtained GQDs was 2.5 nm.

**Figure 3:**
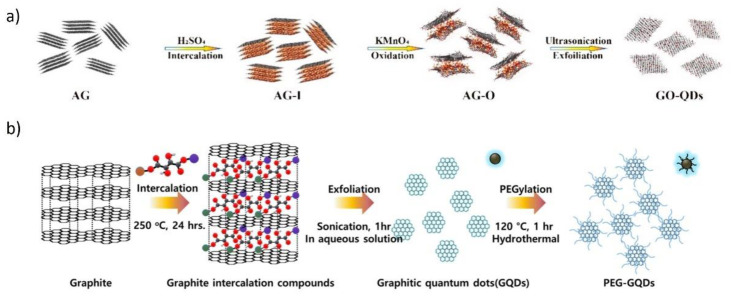
Schematics for the synthesis of graphene quantum dots. (**a**) Chemical intercalation and oxidation of aphanitic graphite. Figure adapted from Reference [111]. Copyright 2020, MDPI AG. (**b**) Hydrothermal synthesis of PEG-GQDs by intercalation and exfoliation of graphite. Figure adapted from Reference [118]. Copyright 2021, MDPI AG.

Shen et al. developed a simple approach for the synthesis of GQDs from aphanitic graphite (Figure 3a) [111]. Aphanitic graphite, H_2_SO_4_, and NaNO_3_ were ultrasonicated at room temperature for 30 min; then, KMnO_4_ was added and it was heated at 35 °C for 40 min. After heating at 98 °C in the presence of DI, the mixture was treated with 30% H_2_O_2_ aqueous solution. After centrifugation, GQDs were collected from the supernatant solution. The product yield was 40 wt.%, the average size was 4.5 nm, and the thickness was ~3 nm (2–3 graphene sheets), while the solubility in water was up to 5 mg mL^−1^. These GQDs emitted intense photoluminescence (PL) in the orange region of the spectrum, with a maximum emission peak localized at 595 nm, under 365 nm light excitation.

The wide range of starting materials, high production yield, and optical properties of the obtained dots are the main advantages of this method. However, the harsh experimental conditions and the use of concentrated acids and strong oxidizing agents make this method environmentally harmful. As can be seen, this could be overcome with the use of alternative, non-toxic oxidizing agents such as H_2_O_2_. Moreover, these approaches require long-term purification after synthesis through a few days of dialysis and centrifugation to obtain a pure product.

#### 2.2.2. Hydrothermal Synthesis of GQDs

Hydrothermal approaches for GQD synthesis include oxidative and reductive cutting starting from carbon materials using high temperature and pressure in a closed environment. In hydrothermal routes, water is the reaction medium.

Kumar et al. reported the facile one-step hydrothermal synthesis of GQDs with tunable emission [113]. GO was dispersed in water by ultrasonication at room temperature for 0.5 h. After the addition of NH_3_, which served as a reducing and stabilizing agent, the mixture was transferred into a Teflon-lined autoclave and heated to 140 °C for 0.5 days. These GQDs, with a ~4 nm average diameter, exhibited violet to green PL.

A new approach was reported by Temerov et al. [114], where GQDs were obtained using graphene capsules as precursors. Graphene capsules were synthesized from graphene-encapsulated metal nanoparticles, which were obtained from lignin and three different transition metal chlorides (Fe, Co, and Ni). After removing the metal nanoparticles, the graphene capsules were mixed with 1M KOH and heated in an autoclave at 250 °C for 12 h. Obtained GQDs with diameters between 15 and 25 nm exhibited a yellowish emission with PL QY of 11.7%, 12.4%, and 12.2%, for GQDs obtained from Fe, Co, and Ni graphene capsules, respectively.

Su et al. synthesized N-GQDs using a GO as a precursor and EDA as a nitrogen source, with a product yield of 20% [115]. GO, EDA, and H_2_O_2_ were dissolved in ultra-pure water and heated in an autoclave at 200 °C for 3 h. The average lateral size of N-GQDs was 1.84 ± 0.28 nm. These GQDs emitted blue light under UV light, with a PL QY of 46%.

Furthermore, Algarra et al. developed an eco-friendly hydrothermal method for GQD synthesis, using 3D graphene materials [116]. In this research, N-GQDs were prepared by mixing a 3D GO and polyvinylpyrrolidone (PVP) in H_2_O and exposing this to hydrothermal conditions at 180 °C for 2 h. Spherical and well dispersed N-GQDs were collected, characterized by a diameter in the range of 2 ÷ 3 nm and a maximum emission peak at 512 nm. To avoid the use of strong oxidation agents and concentrated acids, Lyu et al. presented a thermal-driven advanced oxidation process (AOP) for the synthesis of highly crystalline GQDs with elevated production yields, and functionalized GQDs with tunable PL [117]. In this approach, a GO dispersion in water was added to 30 wt.% H_2_O_2_ aqueous solution. After the addition of FeCl_3_, the mixture was transferred into an autoclave and heated at 180 °C. In the presence of the Fe^2+^/Fe^3+^ redox couple, H_2_O_2_ was dissociated into the hydroxyl radicals (^•^OH). The high oxidative activity of ^•^OH radicals could be responsible for the attacking of defective carbon atoms on the graphene sheets. Under these oxidative conditions, the graphene sheets were cut into small, oxidized GQDs. The production yield of synthesized GQDs was 60 wt.%. The average size of these pristine GQDs (labelled in the original paper as F-GQDs) was ~3.7 nm. These GQDs showed an excitation-independent PL, with a maximum emission peak at 465 nm, and PL QY of 12.9%. Further investigation led to F-GQDs functionalized under hydrothermal conditions to obtain GQDs with different emitted colors. Functionalization of F-GQDs with *m*-phenylenediamine (MPD), EDA, *o*-phenylenediamine (OPD), and *p*-phenylenediamine (PPD) was used to obtain blue (B), green (G), yellow (Y), orange (O), and red (R) GQDs. The experimental conditions were the same as those used in F-GQD synthesis. The average sizes of B-, G-, Y-, O-, and R-GQDs were ~2.7, 3.4, 4.1, 4.5, and 5.1 nm, respectively. Emission peaks were centered at 442, 511, 578, 591, and 620 nm. The PL QYs were 18.3%, 41.4%, 38.3%, 37.5%, and 18.7%. To explore the PL mechanism, bandgap structures of different GQDs were calculated via linear sweep voltammetry, and obtained values were 3.06, 2.50, 2.35, 2.17, and 2.05 eV for B, G, Y, O, and R-GQDs, respectively.

Ji et al. produced poly(ethylene glycol) (PEG)-functionalized GQDs (PEG-GQDs) by graphite intercalation methods [118]. In their approach, graphite was intercalated and exfoliated with sodium potassium tartrate (NaKC_4_H_4_O_6_) under hydrothermal conditions at 240 °C for 24 h. The size of the produced GQDs was controlled by purification using different dialysis membranes (10 and 8kDa NMWL); then, they were PEG-functionalized by mixing with PEG-bis(amine) again under hydrothermal conditions (at 120 °C for 1 h) (Figure 3b). The obtained PEG-GQDs had an average size of 3.23 nm, with narrow size distribution. Oxygen and amide functional groups provided good water solubility.

The hydrothermal synthesis of GQDs allows the one-step synthesis and functionalization of GQDs without the need for complicated post-synthetic treatment. Various starting materials can be exfoliated for a relatively short time under hydrothermal conditions. Obtained GQDs are produced in high yields and possess elevated quantum yields. All these approaches are performed in an autoclave, so there is no real-time control over the reaction’s progression. However, due to the shorter reaction time compared to chemical oxidation and the higher safety, these are the most popular top-down methods for GQD synthesis.

#### 2.2.3. Solvothermal Synthesis of GQDs

**Figure 4:**
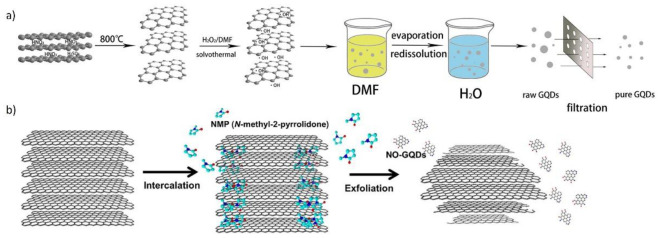
Schematic representation of GQDs prepared by solvothermal method according to (**a**) Tian et al. (Figure adapted from Reference [119]. Copyright 2016, Elsevier B.V.) and (**b**) Kang et al. (Figure adapted from Reference [121]. Copyright 2019, Elsevier B.V.).

Solvothermal approaches in GQD synthesis are very similar to a previously described method. The difference is the use of organic solvents instead of water. A comparison with hydrothermal and solvothermal routes resulted in higher control over GQDs’ properties (size and shape) through fine-tuning of the reaction conditions [106]. A schematic presentation of two solvothermal mechanisms for GQD production is displayed in Figure 4.

Tian et al. developed an eco-friendly, one-step method for GQD production using expanded graphite as a starting material (expanded graphite is modified graphite with a larger interlayer space) [119,120]. Here, DMF was used as a solvent, while H_2_O_2_ acted as an oxidation agent. The dispersion of expanded graphite in a DMF was sonicated for 5 min to remove air between graphite layers. This provided full contact between the solvent and graphite. The reaction mixture was subjected to a temperature of 170 °C for 5 h in the autoclave. The mechanism led to the generation of ^•^OH radicals by H_2_O_2_ decomposition, which served as oxidative scissors for the cleavage of graphite layers. Due to the large interlayer space and extremely high specific surfaces, the dot synthesis reaction rate was accelerated. After purification by filtration, the isolated GQDs showed an average size of 35 nm, high solubility in aqueous medium, and a strong blue emission. The value of PL QY was 15%.

One of the most used solvents in solvothermal methods is NMP, which has similar surface energy to graphite and allows the exfoliation of GQDs. Kang et al. produced highly crystalline edge N/O-functionalized GQDs with high electrical properties using graphite as a starting material [121]. Graphite was dispersed in NMP, placed in a Teflon-lined autoclave, and heated at 300 °C for 24 h. After 5 h of heating and 6 h of drying, pure powder N/O-GQDs was obtained. NMP possesses a high affinity to graphite (contact angle was 28.2°) due to its surface energy in the range of 40–50 mJ/m^2^, which is optimal for graphite exfoliation. When the temperature of the mixture in solvothermal conditions was higher than the NMP boiling point, the vapor pressure of NMP molecules intercalated between graphite sheets led to graphite exfoliation. Moreover, NMP worked as a dopant of N- and O-atoms. Exfoliation started at the edges of graphite and finally terminated with N/O-GQD production. Obtained N/O-GQDs exhibited good dispersibility and colloidal stability. Diameters of dots were 6 ÷ 16 nm and they exhibited blue photoluminescence after laser excitation at 365 nm. The PL QY value was 19.1%.

Shah et al. designed two approaches to obtain fluorescent N-GQDs with distinct optical features, using NMP and DCM [122]. Graphite flakes and ammonium persulphate (AP) were dispersed in NMP and sonicated for 3 h at 50–60 °C. After treatment with H_2_O_2_ at 170 °C, the reaction crude was transferred to an autoclave and heated at 220 °C for 6 h. Next, one part of the sample was extracted with DCM, and the other with water. Exfoliation of graphite by NMP took place according to the previously described mechanism [121]. Before exposure to solvothermal conditions, AP induced the expansion and exfoliation of graphite. Under heating and sonication, the graphite edges and surface adsorbed gases generated as a product of AP decomposition, then diffusing between the graphite layers. During the solvothermal reaction, H_2_O_2_ and unreacted AP performed the oxidative cleavage of graphite layers. As result, oxygen-containing groups were introduced, such as hydroxy, carboxyl, and epoxy moieties. Additionally, NMP and AP served as a source of heteroatoms. These GQDs were labeled as NGQD-d and NGQD-w. Product yields of NGQDs were 46 wt.% for NGQDs-d and 6 wt.% for NGQDs-w. The degree of nitrogen-functional groups was the key factor behind the different optical properties. NGQDs-d with lower content of nitrogen groups exhibited green PL, while NGQD-w with higher content exhibited blue emission. Calculated PL QYs were 63.8% and 26.2%, respectively.

In solvothermal synthesis, depending on the solvent of choice, GQDs exhibit differences in terms of size, structure, and optical properties. In comparison with the hydrothermal approach, this method allows higher control of GQDs’ features.

#### 2.2.4. Electrochemical Oxidation and Exfoliation

The electrochemical synthesis of GQDs is based on the oxidation and exfoliation of carbon starting materials under an electrical field [28]. Solutions of acids, bases, salts, their dispersions in organic solvents, and ionic liquids can serve as electrolytes during the electrochemical process. The lowest applied redox potential was ~1.5 V [123]. During electrochemical synthesis, carbon starting materials can be placed as electrodes (graphite rods [124], carbon nanotubes [125], etc.) or dispersed as electrolytes [126].

Deng et al. proposed the mechanism of electrochemical oxidation [127]. In this work, the starting material was GO dispersed in water without the addition of other chemical reagents. Pt sheets were used both as the cathode and anode. The applied voltage was 15 V and was constant. During this process, the production yield was 65.5 wt.%. Their proposed mechanism is based on the oxidative cleavage of GO by hydroxyl and oxygen radicals generated at the anode during the electrolysis of water. Purification by dialysis was achieved using bags with different pore sizes: 1–3.5 kDa, 3.5–7 kDa, and >7 kDa. Thus, GQDs with different sizes and colors of emitted light were obtained: purple–blue, blue, and green, with average diameters of 2.4 ± 0.3, 3.6 ± 0.2, and 4.6 ± 0.4, respectively.

Electrochemical synthesis in an alkaline solution was described by Xie et al. [128]. They prepared N-GQDs using 3D N-doped carbon nanotube/graphene (N-CNT/N-graphene) as a starting material and nitrogen source at the same time. NGQDs were synthesized in the two-electrode system, where the N-CNT/N-graphene hybrid as a working electrode and a Pt sheet as a counter-electrode were immersed in a NH_3_ solution. The reaction time was 4 ÷ 8 h with a current of 0.01 A and voltage in the range of 5–10 V. This method guaranteed high yields (close to 82 wt.%) and high optical quality (PL QY was ~19.3%) of the obtained dots.

Aqueous solutions of salts also attract attention as electrolytes in the electrochemical synthesis of GQDs due to the better control over the size, shape, optical properties as well as functionalization of the dots. Tan et al. fabricated small-sized red GQDs by the electrochemical cutting of graphene in K_2_S_2_O_8_ aqueous solution at an applied potential of +5.0 V [129]. These GQDs possessed a small uniform size of 3 nm, with pure sp^2^ domains lacking any chemical modifications detected. Due to the small bandgap, the emitted light was shifted to the lower-energy spectral window, i.e., the red region. The aqueous solution of K_2_S_2_O_8_ was a key factor for cutting graphene into small domains. SO_4_^−•^ radicals, generated by the electrochemical reaction of S_2_O_8_^2−^, acted as oxidative scissors, and cut C=C bonds in the graphene structure, producing small sp^2^ structures with oxygen functional groups.

Li et al. produced phosphorus-doped GQDs (P-GQDs) using high-purity graphite rods and Pt as working and counter-electrodes, respectively [130]. Sodium phytate was used as an electrolyte and phosphorus dopant. The reaction lasted for 12 h at a constant voltage of +5.0 V, resulting in quantum dots with a narrow size distribution of 2 ÷ 4 nm.

By combining two different electrolytes but changing the ratio of one of them, GQDs with different structural and optical properties can be obtained. Ahirwar et al. fabricated GQDs and graphene oxide quantum dots (GOQDs) from graphite rods placed as a cathode and anode [131]. Graphite rods were dipped in an electrolyte solution consisting of CA and NaOH dispersions at different concentrations. Obtained GQDs were labeled as GQD_1_, GQD_2_, GQD_3_, and GQD_4_ for CA:NaOH molar ratios of 1:1.5, 1:2, 1:3, and 1:4, respectively. ^−^OH ions generated by water hydrolysis were intercalated between graphite sheets, and oxidized graphite rods at defect sites. Oxygen molecules intercalated between graphene layers and induced their exfoliation. When the content of NaOH increased from 1:1.5 to 1:3, oxidation decreased and GQDs were favored when the molar ratio of CA:NaOH was 1:3. The oxidation was favored in the case of GOQD_4_ because of the large ^−^OH concentration. The structural difference between the four kinds of GQDs caused a variation in their optical properties. GQD_1,_ GQD_2,_ and GQD_4_ exhibited blue and green PL due to the high oxygen content and armchair edges, while GQD_3_ exhibited only blue PL due to the lower oxygen content. The values of bandgaps for GQD_1_-GQD_4_ were 3.55, 3.64, 3.41, and 3.78 eV, respectively.

The electrochemical synthesis of GQDs in a non-aqueous solution implies the use of organic solvents as a medium for electrolyte dispersion [107]. Some of the most used organic solvents are propylene carbonate [126] and acetonitrile [132]. The high boiling point of propylene carbonate (242 °C) [133] can affect their removal from isolated products.

Liu et al. [134] employed ethanol as a solvent due to its non-toxicity. They synthesized GQDs using a three-electrode system and NaOH in ethanol as an electrolyte. Graphite, Pt foil, and Ag/AgCl electrodes were used as the working, counter, and reference electrodes, respectively. The applied voltage was +5.0 V, for 3 h, under a nitrogen atmosphere. During this process, the electrolyte changed color from yellow to brown. The mechanism of the electrochemical oxidation of graphite in water was compared with oxidation in an alcohol environment: Water electrolysis produced gaseous species at the cathode (H_2_) and anode (O_2_). In this reaction, intermediaries such as ^•^OH radicals were generated, leading to the nonselective, fast oxidative cleavage of graphite electrodes, producing large-sized graphene sheets. In alcohol, the activity of ^•^OH radicals can be modulated, allowing the efficient synthesis of GQDs. Under the potential of +3.0 V, synthesis was accomplished within 12 h, while the potential of +7.0 V led to the faster evaporation of ethanol. The average sizes of GQDs fabricated at +3.0 V and +7.0 V applied potential were 2.9 ± 0.3 and 5.2 ± 0.6 nm, respectively. These GQDs showed good dispersibility in aqueous medium and showed blue PL, under 365 nm UV-light irradiation. PL QY values were 9.5%, 11.2%, and 4.6% for GQDs synthesized under potentials of +3.0, +5.0, and +7.0 V, respectively. Based on this research, Jovanović et al. produced GQDs by a similar approach [135,136,137,138]. GQDs were synthesized from graphite electrodes, both working as the anode and cathode [139]. A 3% NaOH dispersion in 96% ethanol served as the electrolyte. At the applied potential of +20.0 V and a current of 20 mA, the electrodes were immersed in the dispersion. After 24 h, the dispersion changed color from pale yellow to brown. Such a color change could be ascribed to the precipitate created by the graphite electrode peeling during the electrochemical reaction. The diameter of the as-synthesized GQDs was 24 nm. These GQDs showed excitation-depended PL in the range of 430–495 nm with a maximum emission peak of 466 nm and 2.07% PL QY.

Electrochemical oxidation and exfoliation of carbon-based materials represent an eco-friendly, fast, and non-toxic way to obtain GQDs. The wide range of starting materials, electrolytes, and electrode materials is the reason for their frequent use by researchers. By setting experimental conditions such as the type of electrolyte, reaction time, applied potential, and ratios of two different electrolytes in the reaction, GQDs with various structural and optical characteristics could be produced.

#### 2.2.5. Microwave and Ultrasonic-Assisted Synthesis of GQDs

The oxidation of starting materials can be assisted with microwave irradiation or ultrasonic waves [140]. Microwave irradiation allows the uniform heating of a reaction medium in which graphene domains with oxygen functional groups introduced by chemical oxidation become more fragile and can be efficiently cut [107].

Li et al. fabricated two-color GQDs by the microwave-assisted chemical cutting of GO [141]. The pristine material was oxidized using a H_2_SO_4_:HNO_3_ mixture under microwave irradiation for 3 h. The mechanism of the reaction lies in the GO epoxy groups’ oxidation by acids, which resulted in the increasing content in carbonyl groups. This caused the graphene domain to become more susceptible to microwave irradiation. Green luminescent GQDs were obtained, and they were turned into blue-emitting GQDs under reductive conditions in the presence of NaBH_4_ at room temperature. It was assumed that GO was partially reduced during the cleavage step. Fourier-transform infrared (FTIR) spectroscopy analysis evidenced the disappearance of the band assigned to epoxy (C-O) stretching vibration at 1260 cm^−1^. The reduction caused changes in ζ-potential, from −58 mV (as detected for pristine GO), −20 mV, and −8 for dots before and after reduction, respectively. Hoang et al. used GO in the ultrafast microwave-assisted hydrothermal procedure for the synthesis of blue-luminescent GQDs [142]. NH_3_ dispersion of GO was heated in a microwave oven at 700 W for only 10 min, returning highly crystalline GQDs with 2 ÷ 8 nm diameter. A reduction in oxygen functional groups and the introduction of N-containing groups was confirmed by X-ray diffraction (XRD) and FTIR spectroscopy. This method is considered an ultrafast, low-cost, energy-safe, and non-toxic technique for GQD synthesis. Microwave-assisted acidic oxidation of fluorinated graphene oxide (FGO) was used for the production of fluorinated GQDs (F-GQDs) [143]. A mixture of FGO, H_2_SO_4_, and HNO_3_ was refluxed under microwave irradiation at 650 W for 6 h. The presence of F in the sample was confirmed by X-ray photoelectron (XPS) spectroscopy, with F-atoms detected as C-F groups. A lower F/C ratio was detected in F-GQDs in comparison to FGO. These F-GQDs showed bright blue photoluminescence with a PL QY of 7.5%.

In ultrasound-assisted approaches, a key factor for carbon material cutting is the creation of bubbles, which create pressure between the layers of the starting material. The rapid flow of the liquid medium under ultrasonic conditions and strong hydrodynamic forces contribute to exfoliation [140].

Considering the need for non-toxic methods in GQD synthesis, an O_3_/H_2_O_2_/ultrasound system was prepared for the first time by Wen et al. [33]. A mixture of GO water suspension and 30% aqueous H_2_O_2_ was constantly purged with O_3_ for 3 h under ultrasonic irradiation at 150 W. The proposed mechanism consists of two steps: (i) C=C and C-C bond oxidation is induced by ^•^OH radicals generated under ultrasound irradiation, where ultrasound increases the ^•^OH amount and encourages molecular vibrations that contribute to GO cutting; (ii) ^•^OH interaction with the GO surface, oxidizing C-O and C=O groups and scissoring GO fragments in GQDs, with -COOH functional groups. The presence of these groups in the sample was confirmed by XPS and FTIR spectroscopy. Obtained GQDs possessed a strong PL emission peak localized at 520 nm. Another green method for GQD synthesis was developed by Gao et al. [144]. They fabricated GQDs by the ultrasound-assisted exfoliation of graphite and their derivates in supercritical CO_2_/H_2_O conditions. By a combination of ultrasound and supercritical liquid, the efficient stratification of layered graphite materials (pristine graphite, expanded graphite, and graphite oxide) was achieved. In this case, the effects of the water amount, pressure in the system, and ultrasonic power on the GQD yield were investigated, confirming that the highest production yield was achieved under the lowest content of water (20%), the highest applied pressure (20 MPa), and the highest ultrasonic power (2 KW). The impact of the hydrophobicity/hydrophilicity of the starting material on GQD synthesis was explained considering that hydrophobic materials such as pristine graphite are more susceptible to bubbles generated in ultrasonic conditions, while hydrophilic materials such as GO are almost unchanged.

Microwave-assisted methods significantly shorten the reaction time, which is the main advantage of this method. Herein, the staring material is uniformly heated and it is easily cleaved. The use of strong acids for pretreatment could be a relevant disadvantage. In the ultrasonic approach, ultrasonic power can affect the production yield of the obtained GQDs. This represents an easy, low-cost, and eco-friendly method that enables the rapid preparation of products.

#### 2.2.6. Laser Pulse-Assisted Synthesis

Liquid-phase laser ablation (LA) is a single-step, eco-friendly method for GQD synthesis due to the short reaction time and the absence of toxic compounds and byproducts of the reaction. Using a laser beam in a liquid phase, GQDs were synthesized. Two main mechanisms of the laser-assisted synthesis of GQDs are reported in the literature: thermal evaporation and explosive ejection. In thermal evaporation, during contact, a laser pulse with a solid target, a high temperature, and high-pressure plasma is created at the solid–liquid interface. This plasma expands adiabatically and condenses, forming a cluster. In another suggested mechanism, explosive ejection, laser irradiation causes the melting of the target material, generating nanodroplets. These nanodroplets are launched into the liquid, where nanoparticles are formed [145]. The target materials for laser ablation include multiwall carbon nanotubes (MWCNTs) [146], graphite powder dispersion [147], highly oriented pyrolytic graphite (HOPG) [102], graphene oxide [148], or carbon black [149].

Calabro et al. fabricated GQDs using carbon nano-onions as a starting material in DI under a laser pulse [145]. Pellets of nano-onions were dispersed in the aqueous medium and exposed to a laser pulse width at 532 nm of 5–7 ns with 10 Hz repetition frequency. The ablation was carried out at 1.30 W for 7 h. Compared with GQDs obtained by the traditional chemical oxidation of carbon nano-onions (CO-GQDs), GQDs produced by LA exhibit a smaller diameter (4.1 nm and 1.8 nm, respectively). According to XPS and FTIR spectroscopies, considerable amounts of sp^3^-cabon atoms are present despite sp^2^-carbons and hydroxyl groups being more abundant than carboxyl ones. The presence of hydroxylic groups caused a blue shift in the emission band in the PL spectra of GQDs produced by LA.

Laser ablation can be combined with the traditional methods to improve GQDs’ properties. Leon et al. fabricated N-GQDs by a combination of a laser pulse and solvothermal methods [150]. Cryomilled graphite pellets were exposed to a laser beam (pulse width = 10 ns, pulse repetition rate = 100 Hz). The obtained dispersion was exposed to solvothermal conditions at 65, 90, and 120 °C for 72 h, resulting in the production of GQDs characterized by increasing PL intensity after solvothermal treatment, showing QY PL values of 0.60, 0.91, 1.74, and 4.05% for the GQDs before and after solvothermal treatment at temperatures of 65, 90, and 120 °C, respectively.

Laser ablation is the non-toxic synthesis of small-sized GQDs within short reaction times. This method can be either performed alone or can be combined with other approaches in order to obtain higher-quality products.

## 3. Characterizations

Investigation of the morphological, structural, chemical, and optical properties of GQDs is performed by various microscopic and spectroscopic techniques: transmission electron microscopy (TEM), scanning electron microscopy (SEM), atomic force microscopy (AFM), Raman spectroscopy, FTIR, XPS, XRD, UV–visible spectroscopy, and PL spectroscopy [151]. In the following section, the most frequently used techniques and obtained results are discussed.

### 3.1. Morphology Investigation

**Figure 5:**
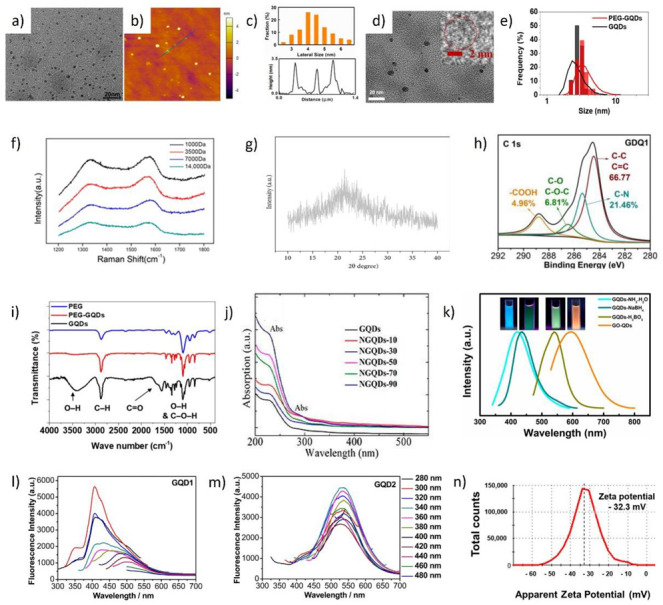
Methods for GQD characterization: (**a**) TEM picture, (**b**) AFM picture, (**c**) size and height distribution. Figures adapted from Reference [111]. Copyright 2020, MDPI AG. (**d**) HR-TEM picture of GQDs. Figure adapted from Reference [154]. Copyright 2018, MDPI AG. (**e**) DLS of GQDs. Figure adapted from Reference [118]. Copyright 2021, MDPI AG. (**f**) Raman spectra of GQDs (1, 3.5, 7, 14 kDa), with detailed view of the D and G bands. Figure adapted from Reference [158]. Copyright 2021, MDPI AG. (**g**) XRD pattern of GQDs. Figure adapted from Reference [142]. Copyright 2020, Hindawi Publishing Corporation. (**h**) XPS spectrum of GQDs, C 1s band deconvoluted to four different bands corresponding to functional groups with percent. Figure adapted from Reference [154]. Copyright 2018, MDPI. (**i**) FTIR spectra of PEG, PEG-GQDs, and GQDs. Figure adapted from Reference [118]. Copyright 2021, MDPI AG. (**j**) UV–visible spectra of GQDs and N-GQDs. Figure adapted from Reference [162]. Copyright 2017, MDPI AG. (**k**) PL spectra of GQDs-NH_3_·H_2_O, GQDs-NaBH_4_, GQDs-H_3_BO_3_, and GO-QDs with photographs of emitted colors under an excitation wavelength of 365 nm. Figures adapted from Reference [111]. Copyright 2020, MDPI AG. (**l**) PL spectra of GQD1 (excitation-dependent PL, red-shifted maximum) under λ_exc_ 280–480 nm. (**m**) PL spectra of GQDs (excitation-independent PL, stable maximum at 535 nm), under λ_exc_ 280–480 nm. Figures adapted from Reference [154]. Copyright 2018, MDPI AG. (**n**) ζ-potential measurement of GQDs. Figure adapted from [118]. Copyright 2021, MDPI AG.

To investigate the size and the shape of GQDs, TEM has been used in many studies. TEM offers information about morphology, and it is often used to calculate the size distribution of GQDs (Figure 5a,c) [109,112,114,152,153]. High-resolution (HR-TEM) allows the investigation of the microstructure of GQDs, such as crystallization, lattice fringes, etc. [154]. Using HR-TEM, it is possible to measure d-spacing (distance between planes of atoms), which can help to confirm the graphite structure in the investigated sample [112,115,117,131,143,152]. Figure 5d shows an HR-TEM image of GQDs obtained by combining the oxidation of GO in the presence of HNO_3_ and hydrothermal conditions (200 °C, 8 h) [154]. The size of the GQDs was in the range of 2–5 nm. HR-TEM can be combined with fast Fourier transformation (FFT), which improves the examination of GQDs’ crystallinity [121]. The surface morphology and topography of samples is usually examined by SEM [119,151]. AFM is most commonly applied for measuring the height of GQDs. These data can be used to estimate the number of graphene layers. An example of a GQD height distribution histogram is presented in Figure 5c [111]. For the investigation of GQDs’ hydrodynamic diameter, dynamic light scattering (DLS) is a frequent choice (Figure 5e) [118].

### 3.2. Chemical Composition and Surface State Investigation

The chemical composition and structure of GQDs can be investigated using different techniques: Raman and FTIR spectroscopies, XPS, and XRD are standard techniques, but H- and ^13^C-NMR spectroscopy, mass spectroscopy, electron paramagnetic resonance (EPR), and elemental analysis are used as well [135,155,156].

In the Raman spectrum of GQDs, two bands around 1367 cm^−1^ (D band) and 1529 cm^−1^ (G band) are two distinctive features. The G band stems from the sp^2−^ carbon network while the D band is a result of defects in the graphite structure. The values of intensity ratio between the D and G bands (I_D_/I_G_) indicate defects in graphene structures [112,114,117,152,157]. For example, in the Raman spectra of four GQDs separated using dialysis tubes filtering different molecular weights (1, 3.5, 7, and 14 kDa), I_D_/I_G_ ratios for the different samples were 0.98, 0.93, 0.94, and 1, respectively (Figure 5f) [158].

The basic technique used for investigating a disturbance in graphitic structures as a consequence of the cleavage of a carbon starting material during GQD synthesis is XRD. The XRD pattern of pure graphite consists of a single, strong peak centered around 26°, corresponding to the (002) plane. After cutting a graphite material during synthesis, the GQDs’ XRD pattern presents a broad peak that indicates disorder in the graphene structure [112,117,122,129]. In Figure 5g, an XRD pattern of GQDs is presented. Only a broad peak at 21.7° is detected, and it is shifted with respect to the graphite signal, due to the increase in the interlayer spacing to 0.403 nm from 0.335 nm in graphite [142].

XPS is often used for the examination of GQDs’ chemical composition and functional groups. An XPS survey of GQDs reveals two principal peaks, localized at 284 eV and 532 eV. The first one corresponds to carbon (C 1s) and the latter to oxygen (O 1S). After deconvolution, peaks are assigned to sp^2^/sp^3^ bonds (284.76 eV), C-O (286.5 eV), O-C=O (288.5 eV), C-OH and O-C-O (533.0 eV), and C=O (531.7 eV) [119,122]. In N-GQDs, a strong peak at 400 eV is present, which corresponds to the presence of nitrogen (N 1s). N-atoms can be bonded to C-atoms in four different ways, pyrolytic, pyridinic, graphitic, and oxidized, with peaks at 399.8, 398.2, 401.3, and 402.9 eV, respectively [121,157]. Moreover, the percentage of each atom can be calculated by XPS analysis, returning XPS as a standard technique to quantify the dopant’s presence within the GQD lattice [117]. Deconvolution of the C 1s band at four different peaks is presented in Figure 5h, and they were assigned to C=C/C-C (~284.5 eV), C-O-C (~286.5 eV), -COOH (~287.8 eV), and C-N (~285.6 eV) bonds. In addition, the percentage of these groups was 66.77, 6.81, 4.96, and 21.46%, respectively [154].

In the FTIR spectrum of GQDs synthesized by the electrochemical oxidation of graphite electrodes, one can observe a band at 3421 cm^−1^ that stems from the vibration of the O-H stretching mode in hydroxyl groups attached to carbon atoms [159]. Other bands located at 2970, 2930, and 2877 cm^−1^ correspond to vibrations of the C-H bond in hydrocarbon fragments. Moreover, bands at 2697 and 2781 cm^−1^ originate from bonds in aromatic aldehydes [139]. The band at 1700 cm^−1^ stems from the C=O bond in the carboxyl group. The band around 1570 cm^−1^ is assigned to the C=C bond in aromatic domains. The bands at 1010 and 1365 cm^−1^ correspond to the C-O bond and symmetric stretching of the O-C=O group, respectively. In the FTIR spectra of γ-irradiated and amino-functionalized GQDs, a band at 3360 cm^−1^ is observed and results from N-H bonds in the GQDs’ structure [139]. In phosphorus-doped GQDs, observed bands at 1198 and 1450 cm^−1^ stem from the stretching vibration of P-O and P-C bonds, respectively [130]. Nitrogen- and boron-doped GQDs possess specific bands at 1355 cm^−1^ that correspond to stretching vibrations in C-N bonds, and at 1432 cm^−1^, which is attributed to the stretching vibration of B-O [157]. In the FTIR spectrum of sulfonated GQDs, bands at 1130 and 1215 cm^−1^ arise from the symmetric and asymmetric stretching vibration of a S=O bond in -SO_3_H [160]. A characteristic band in the FTIR spectrum of the fluorinated GQDs appears at 1220 cm^−1^ and is attributed to the vibration of the C-F bond [143]. Figure 5i shows the FTIR spectra of PEG, PEG-GQDs, and GQDs. As can be seen, in the FTIR spectrum of PEG-GQDs, the carboxyl group at 1660 cm^−1^ completely disappears in comparison with the GQDs’ spectrum [118].

The surface charge can be also investigated by measuring GQDs’ ζ-potential, depending on the presence/absence of charged groups. The ζ value of PEG-GQDs was −32.3 mV (Figure 5n) [118], and it can vary from −34.6 to +9.1 eV [139].

### 3.3. Optical Properties Investigation

GQDs show strong absorption in the UV region of the spectrum and photoluminescence in the visible part of the spectrum [106,161]. In GQDs’ UV–visible absorption spectra, the peaks at 360 nm correspond to the n-π* transition of the C=O bond, and features at 280 or 230 nm are attributed to the π-π* transition of aromatic sp^2^-carbon atoms [112,114,115,121]. In some cases, these peaks can be shifted to 301 and 228 or 290 nm for the n-π* transition of C=O and the π-π* transition of the sp^2^-aromatic fragment, respectively. This results from functionalization with heteroatoms or it is due to the presence of functional groups [122,157]. Changes in peak positions can be attributed to changes in the chemical environment around the chemical bond [139]. For multicolored GQDs (B-, G-, Y-, O-, and R-GQDs) obtained by functionalization with different nitrogen sources and described earlier [117], the same absorption peak on 280 nm was observed. This peak seems related to the π-π* transition of C=N bonds. Moreover, the absorption intensities increase from B-GQDs to R-GQDs due to the increasing content of nitro-substituted aromatic rings. Moreover, absorption peaks in the visible-light region are revealed at 381, 410, 450, 518, and 580 nm for B-, G-, Y-, O-, and R-GQDs, respectively. These GQDs show excitation-dependent PL. In Figure 5j, the UV–visible absorption spectra of pristine GQDs and GQDs produced from N-doped GO through a photochemical route during different periods are shown. GQDs and N-GQDs show strong absorption peaks at 240 nm, which correspond to the π-π* transition in the sp^2^-structure. N-GQDs show an absorption peak at 300 nm, which usually occurs in nitrogen-functionalized GQDs [162].

GQDs’ PL has not been sufficiently investigated to establish a theory of their origin, so there is a lack of fundamental knowledge that needs to be resolved. It is assumed that GQDs’ PL arises as a result of several combined factors: the quantum confinement effect, edge effect (zig-zag or armchair edge), radiative recombination of electron–hole pairs, functional groups on their surface, and vacancy defects. The PL of GQDs can be tuned by setting a specific synthetic design or using post-synthesis treatments that result in the introduction of either heteroatoms or functional groups or both [106]. Thus, GQDs emit a different colored light [127,128,129]. In Figure 5k, PL spectra of GQDs functionalized with NH_3,_ NaBH_4_, and H_3_BO_3_ are presented. As can be seen, four kinds of GQDs (GQDs-NH_3_·H_2_O, GQDs-NaBH_4,_ GQDs-H_3_BO_3,_ and GO-QDs) exhibit a maximum emission peak at different wavelengths, 425, 435, 539, and 595 nm, emitting four different colors (Figure 5k, inset) under an excitation wavelength of 365 nm [111]. In the literature, two kinds of PL behavior were reported: a more frequent excitation-dependent [115,116,117,121,122,127,131,139,154] and, more rarely, excitation-independent behavior [129,154]. In the first case, the peak position on emission spectra depended on the excitation light wavelength. Excitation dependence can be attributed to electron-conjugated structures, zigzag sites, and emissive traps. Various functional groups have different energy levels and influences on PL emission. Moreover, the size distribution causes differences in bandgaps. According to the quantum size effect, with increasing GQD size, the bandgap decreases and allows emission at high wavelengths [114]. Excitation independence implies the unshifting of the emission peak under different excitation wavelengths and can be attributed to a stable surface state [157] and uniform size of GQDs [129]. PL spectra of GQD_1_ and GQD_2_ are presented in Figure 5l,m. Under excitation wavelengths in the range of 280–480 nm, the maximum emission peak of GQDs_1_ was red-shifted from 480 to 500 nm, while the maximum of GQD_2_ remained unchanged at 535 nm. Differences in PL behavior between GQD_1_ and GQD_2_ were attributed to differences in size and surface state (O- and N-atoms abundance), which leads to bandgap changing [154].

## 4. GQD Applications in Energy-Related Applications

The increasing global energy demand is currently threatening the health of our society: fossil fuels still play a crucial role in the production of electricity and, considering the limited availability and the localization of such resources in very specific areas of the globe, a combined social and geopolitical crisis is emerging. The reliance on fossil fuels has fueled the introduction into the atmosphere of massive amounts of greenhouse gases—CO_2_, amongst others—posing risks to the health of the whole planet, as critical and extreme events such as disastrous global warming are being observed. This situation can only be defined as a climate emergency [163]. The call for decisive action is urging scientists to devote huge efforts to the development solar energy technologies; as a uniformly distributed, abundant, green vector of energy, the sun [164,165] could help humanity to limit its further negative effects on the environment. The development of solar technologies is an important solution, moving in the right direction [166], but photovoltaic devices must be developed to be not only efficient but cheap, and they must be based on materials whose production is environmentally sustainable. To this aim, GQDs could be the cornerstone of this technology, considering their production methods, which are affordable and can be based on poor and accessible biomasses (Section 2.1.5); moreover, they possess solution processability and electric and optical features that make them prominent candidates for this technology. GQDs have been tested in several photovoltaic architectures, such as perovskite solar cells [167], copper indium gallium (di)selenide (CIGS) solar cells [168], and others, but dye-sensitized solar cell (DSSC) technology is the research field in which graphene quantum dots have found the most significant application and investigation. For this reason, Section 4.1 will offer a detailed overview of the most recent literature focusing on the exploitation of GQDs in DSSC fabrication.

If we shift our attention away from optical properties, previous studies on GQDs indicated that the charge transport phenomena are dominated by the charge storage in the trapping levels [169], thus suggesting their usefulness in energy storage devices, a technology that is becoming of fundamental importance as its coupling with solar cells is necessary in order to achieve an efficient system, able to supply energy without any time and irradiation restrictions, working as a buffer network when power production lowers but energy needs remain high. On these premises, Section 4.2 will offer an overview of the most recent literature on capacitors and supercapacitors, with the aim of developing a portfolio of GQDs able to be used also in this important branch of electronics, which will strongly support the evolution of energy networks.

### 4.1. GQDs as Active Components in DSSCs

**Figure 6:**
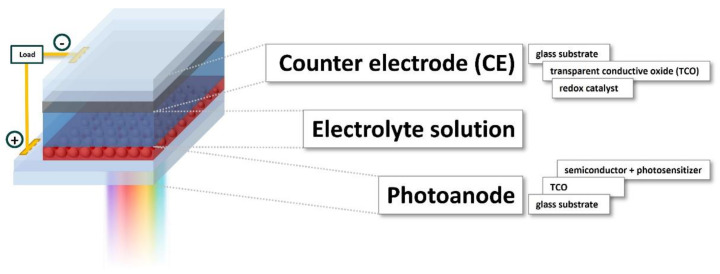
Schematic representation of DSSC’s architecture. Due to their features, GQDs can be potentially applied as constituents of all three of the main components of DSSCs to further boost their performance.

Dye-sensitized solar cells (also Grätzel cells) are a family of thin-film photovoltaic devices [170,171,172,173]. Their history dates back to the end of the previous century, when O’Regan and Grätzel fabricated an innovative, cheap photovoltaic system based on colloidal titania’s photochemical properties [174]. The fabrication scheme (reported in a simplified fashion in Figure 6) can guide the reader to understand the key issues behind the cell’s operation: it is based on the presence of a thin layer, where a semiconductor (usually TiO_2_) is mixed with a dye. (i) When the dye is excited by sunlight, it will transfer the excited electron to the conduction band of the semiconductor. (ii-a) This electron will diffuse through the semiconductor, where it will be collected by an electrode and flow through the external circuit to the counter-electrode (CE). (ii-b) At the same time, the oxidized dye will be reduced by the electrolyte, regenerating its ground state. (iii) The electron arriving at the CE will be given to the oxidized redox mediator, regenerating the initial situation.

Thus, DSSCs are simply photoelectrochemical cells whose advantages can be summarized as follows: (i) low cost of the starting materials, (ii) well-established mechanism, (iii) achievable integration with industrial printing techniques, (iv) (semi)transparency and (semi)flexibility, with facile integration of the final devices into building façades, windows, etc., and (v) robustness. Their performance can be measured using several recognized parameters, such as power conversion efficiency (PCE), open-circuit voltage (V_OC_), short-circuit current (J_SC_), and fill factor (FF), parameters that can be improved by enhancing the design of the cell, employing new parameters, and focusing on the light-harvesting system (here, GQDs are some of the most investigated materials).

However, even ignoring the higher efficiencies that characterize the most popular Si-based technology, the use of the liquid electrolyte is a major concern, as the volatile medium requires appropriate cell sealing strategies. Moreover, electrolytes can create environmental concerns, and precious metals are usually employed at the CE. Nevertheless, these key issues have been recently addressed using solid-state or quasi-solid electrolytes in the first case and innovative materials in the latter case, and GQDs are playing a role in this direction, as will be discussed in the following paragraphs.

#### 4.1.1. GQDs as (Co)Sensitizers in DSSCs

Due to their electronic properties and the possibility to opportunely modulate them during the synthetic step, GQDs can be usefully exploited in DSSC fabrication as the material of choice for different components. The simplest integration strategy takes advantage of GQDs’ optical properties; there are a number of reports in the literature in which they act either as sensitizers or co-sensitizers. The latter choice consists in the combination of GQDs with another dye; this is a popular approach as there is a long history of well-consolidated knowledge on fully organic dyes or coloured transition metal complexes, particularly Ru-species. GQDs in combination with another dye can provide both extension of the spectral absorption range and improved charge carrier generation/propagation mechanisms. Among several studies, Mihalache et al. applied GQDs as co-sensitizers in DSSC architectures [175]; starting from the synthetic design, they modulated the materials to obtain good electronic matching between the TiO_2_/GQDs/N719 Ru-dye assembly; compared with benchmark devices where GQDs were the sole sensitizers, the final architecture allowed the authors to obtain a better-performing cell, where the combined chromophores guaranteed the extension of the spectral absorption range, thus bypassing the presence of strong absorption by the so-synthesized GQDs simply in the UV spectral range. The device further benefitted from the efficient energy transfer between GQDs, the satisfactory spatial separation of the electrons and holes, the improved electric characteristics due to the electron injection from the dye to TiO_2_ and GQDs, and longer recombination times. In another approach, Maity et al. focused on the coupling of poly(aniline) and GQDs at different wt.% ratios, to realize DSSCs in combination with commercial Ru-based N719 dye [176]. The best DSSC design returned a device with 3.12% PCE; such performance was attributed to the superstructure formed by the interaction between poly(aniline) and GQDs, resulting in the formation of cylindrical lamellae with a large conjugation length, and the GQDs residing between the polymer chains. These features contribute to the prolonged lifetime values of the photo-injected electrons, thus supporting the interesting performance of the device. Although the use of poly(aniline) is valuable in terms of the realization of a fully organic device, better results were obtained by Salam and collaborators [177], with the decoration of more traditional, nanostructured TiO_2_ with GQDs, which showed improved N719 dye absorption, more efficient electron extraction of the GQD hybrid in comparison with pure TiO_2_, and an overall more efficient PCE of the fabricated solar cells compared with the reference cell with no carbon nanomaterial (6.22 vs. 4.81%, respectively). Subramanian and co-workers took a step further in the optimization of the N719-based designs, optimizing the synthesis of graphene quantum dots with appropriate energy levels [178]. In their design, precise control over GQDs’ synthetic conditions allowed them to isolate the material able to act as an efficient energy donor to the N719 dye by means of Förster resonance energy transfer (FRET), as suggested by spectral evidence, and they suggested a molecular interaction supporting the effective energy transfer. Experimental data returned a FRET efficiency high as 27%, and the DSSCs based on this N719-GQD combination possessed a PCE of 7.96% and a J_SC_ of 16.54 mA cm^−2^, which was 30% higher than a benchmark cell with no GQDs as co-sensitizers. These results are confirmed by a similar work published slightly later [179], where the authors documented a different synthetic approach and fabrication technique, although referring to a very similar design of the device. GQD doping can be an intriguing and efficient strategy to further tune their optical and electronic properties when the synthesis is performed. A report from 2020 focused on the synthesis of N-GQDs [180], which resulted in a homogeneous diameter (ca. 9 nm), good dispersibility, and intense orange luminescence in the visible spectral window, with maximum emission centered at 590 nm. Application in DSSC technology revealed the central role played by GQDs in providing efficient charge separation and collection, probably ascribed to the correct energy level alignment with N719 dye and TiO_2_. Final DSSCs were characterized by increased J_SC_ (up to 17.65 mA cm^−2^) and 7.49% PCE in the best case.

**Figure 7:**
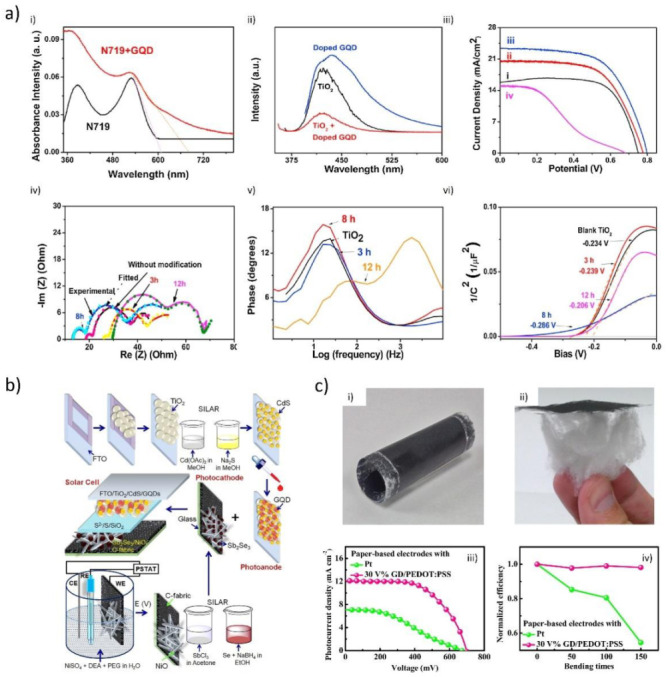
GQDs in DSSC architectures. (**a**) Collection of (**i**) UV–visible spectra of N719 dye with and without N,F,S-doped GQDs and (**ii**) of PL spectra of N,F,S-doped GQDs, and TiO_2_ with and without N,F,S-doped GQDs. (**iii**) Variation in I–V characteristics of DSSCs α) with TiO_2_/N719 dye photoanode and β-δ) with different N,F,S-doped GQDs integrated into the photoanode at different loading ratios. (**iv**) Comparison of EIS of TiO_2_, N,F,S-doped GQDs-TiO_2_ at three different loading ratios. (**v**) Bode plot of the respective EIS and (**vi**) Mott–Schottky analysis of the previously analyzed devices under forward bias under dark conditions. Figures adapted from Reference [181]. Copyright 2017, Elsevier B.V. (**b**) Schematic representation of the fabrication strategy adopted for the preparation of the photoanode, photocathode, and the complete DSSC device. Figure adapted from Reference [186]. Copyright 2017, American Chemical Society. (**c**) Paper-based counter-electrodes made with 30 vol.% GQDs/PEDOT:PSS composite demonstrate that the material is (**i**) flexible and (**ii**) light-weight. (**iii**) The J-V curves of the flexible DSSCs using traditional Pt CE and the GQDs integrating composite. (**iv**) Normalized efficiency of the flexible DSSCs using the two types of paper-based counter-electrodes after being bent up to 150 times. Figure adapted from Reference [191]. Copyright 2017, Elsevier B.V.

The threshold of 10% PCE devices was finally extended by Kund et al., who produced DSSCs with 11.7% PCE and FF of 71% after decorating a TiO_2_ layer with N,F,S-doped GQDs [181]. The synthetic approach aimed at introducing N-atoms to reduce the bandgap, S-species to facilitate the electron transfer, and F-atoms to enhance the stability of the hybrid material by hydrogen bonding with the -OH groups of the TiO_2_ nanoparticles. The study revealed that the dots improved the electron transport mechanism and contributed to the suppression of the electron recombination mechanisms; they also led to high stability that guaranteed PCE values around 10% even for devices prepared one month before the measurements were performed (see Figure 7a).

In a recent example [182], hybrid photoanodes for DSSCs were fabricated by combining ZnO decorated with GQDs and zinc tetrakis(4-carboxy phenyl)porphyrin (TCPPZn) as a principal sensitizer; the Zn porphyrin was chosen due to the presence of a LUMO orbital in good overlap with the ZnO conduction band, thus funneling electron injection into the semiconducting oxide. Studies investigated combinations based on different amounts of GQDs, with the best fabricated DSSCs prepared in the presence of GQDs at a 40 wt.% ratio (J_SC_ = 10.1 mA cm^−2^, V_OC_ = 0.48, PCE = 2.45%, FF = 0.51); similar to the previous examples with N719 dye, time decay measurements revealed evidence of the FRET mechanism, which boosted the electric properties. Although the absolute PCE value was far from those of previous devices, this is an interesting example based on a very simple processing approach and low-cost starting materials. The need for low-cost and greener approaches led Saedi et al. to investigate a design in which natural dyes extracted from red (*Gracilaria*) and green (*Ulva*) algae were considered as photosensitizers [183]. In this example, a DSSC photoanode was fabricated starting from porous TiO_2_, which was later decorated with GQDs, with the natural dye absorbed to form this hybrid material. It is important to immediately state that, even considering the best-performing cell based on TiO_2_/GQDs/*Gracilaria*, the performance remains quite poor, with PCE = 0.94%, J_SC_ = 2.26 mA cm^−2^, V_OC_ = 0.73 V, and FF is 56%. However, the study suggests that there are many biomasses that are readily available and accessible, and an important technology such as photovoltaics can be based on very poor but naturally abundant products.

The combination of GQDs with organic dyes has been proven to be a popular strategy, but several studies moved away from this route, looking for better-performing and stable architectures. It has been reported that CdSe quantum dot-sensitized solar cells could benefit from the introduction of GQDs as co-sensitizers [184]. In this case, GQDs mainly acted as a charge recombination suppressor, as electron impedance spectroscopy and transient photovoltage decay measurements confirmed; after optimizing the GQD coating deposition in terms of deposition sequence, concentration, and time, the final devices offered an improved PCE of 6.59% under AM 1.5G full one sun illumination. Recently, PL quenching and lifetime measurements revealed that, in the case of GQDs/CdSe quantum dots, it is also possible to identify the FRET mechanism, so as to gain deeper knowledge of the system, aiming at further performance improvements [185]. Similar results were recently reported by Kolay and collaborators for a different design centered around CdS quantum dots as the main photosensitizer [186]; the performance was similar in terms of PCE, although the design was more complex in terms of the fabrication protocol. In a very recent research report, N-GQDs were synthesized according to a hydrothermal method and then combined in a TiO_2_/GQDs/CdSe quantum dots assembly to fabricate DSSCs [187] (see Figure 7b). Here, N-doping provided a co-sensitizer able to efficiently transport electrons and suppress charge recombination phenomena, providing further long-term stability to the so-designed photoanode. Improved performance was revealed, with the best solar cell characterized by J_SC_ = 18.80 mA cm^−2^ and a PCE increasing by 25% compared to the benchmark cell with no GQDs.

Even if the coupling of GQDs with another dye provides notable results in DSSC fabrication, it would be desirable to simplify the design and rely on a single light-harvesting species. For this reason, very recent years saw mounting interest towards GQDs as a stand-alone dye; for example, Sharif et al. managed to study the preparation of sensitized titania starting from GQD solutions at different concentrations [188]. In general, they observed for all the designs increased light absorption in the 375–600 nm spectral range, which resulted in Jsc (15.49 mA cm^−2^) and PCE (6.97%) for the best sampled cell, which is a notable increase if compared with the values measured for the reference design with no GQDs (12.10 mA cm^−2^ and 4.08%). The explanation for this increasing performance is related to the role played by GQDs acting as a carrier recombination suppressor, efficient charge carrier transporter, and light sensitizer, compensating for the intrinsic limits of TiO_2_ in terms of electron transport properties and the light absorption range limited to the UV window.

#### 4.1.2. Counter-Electrodes and Electrolytes Based on GQDs

GQD applications are not limited to the active electrode in DSSCs, but several research teams focused their attention on the CE, considering that the elevated electric properties and elevated stability should return interesting results. The search for new, low-cost, and stable materials is focused on finding candidates to replace the classic Pt electrode. With this purpose in mind, Wang and Lu reported the preparation of a composite material made of carbon aerogel (CA) decorated with GQDs [103]. In their approach, the advantageous combination of a material with a high specific surface area (CA) and a partner with elevated catalytic efficiency (GQDs) produced a composite able to reduce I_3_^−^ to I^−^ as efficiently as Pt but with superior long-term stability, as confirmed by cyclic voltammetry measurements; the cathodic current dropped significantly after 500 cycles for the Pt, whereas it slightly increased for the GQD/CA electrodes. Similarly, the work by Chang and others pursued the concept of a support with a high surface area, decorated with GQDs [189]: in their case, graphene foam nanosheets were mixed with the dots, demonstrating high electronic conductivity and fast electron injection. The optimized DSSCs showed an appealing 9.59% PCE, with V_OC_, J_SC_, and FF of 0.75 V, 19.23 mA cm^−2^, and 66.5%, respectively. These data appear more interesting when compared to reference cells prepared with the same design except for the CE, made of either Pt or simple graphene foam: looking at the PCE, the value for the design integrating the latter CE was a negligible 0.68%, but, in the presence of PT CE, the measured value (8.58%) was inferior to the recorded value in the presence of GQDs of 12%.

Moving away from carbonaceous nanomaterials, counter-electrodes based on poly(3,4-ethylenedioxythiophene):poly(styrenesulfonate) (PEDOT:PSS, a very popular material in solar cell technology) benefitted from a GQD dispersion as well [190]. In the work reported by Lee et al., electrodes prepared with GQDs possesses a rough surface morphology, high conductivity, and electrocatalytic activity, as well as low charge transfer resistance towards the I_3_^−^/I^−^ redox reaction; moreover the cell efficiency was improved to a 7.36%, which is much higher than the 5.14% recorded for the reference design with PEDOT:PSS traditional CE. The same authors offered proof of the CE mechanical stability once applied to flexible DSSCs [191], where the performance was retained after folding the electrode 150 times, while the standard Pt electrode lost 45% of its performance after such manipulation (see Figure 7c). There is a report in which a CE produced by the electropolymerization of poly(aniline) in the presence of GQDs is described [192], but poor results in terms of PCE (although superior to the reference cell with no quantum dots) make this example of limited relevance. In recent years, a lot of interest has been devoted to a hybrid electrode made of electrically conductive perovskite strontium ruthenate (SrRuO3) decorated with GQDs [193]. The inorganic matrix was chosen as the starting material for the construction of CEs due to its electric conductivity and high catalytic activity. Porous matrices were prepared starting from SrRuO_3_, which was then dispersed in a binder solution (ethyl cellulose and terpineol in ethanol) to obtain a paste that was finally spin-coated on conductive glass; this passage is not trivial, because it guarantees high porosity compared to sputtered SrRuO_3_ films, which allows better decoration with GQDs once the perovskite is dipped into GQD stock solution, ultimately guaranteeing improved catalytic activity towards the I_3_^−^/I^−^ redox reaction. Cyclic voltammetry (CV), electrochemical impedance spectra (EIS), and Tafel polarization measurement allowed the authors to check the quality of the electrodes, and final characterization of the DSSCs showed that the reference device with sputtered a SrRuO_3_ film CE had a PCE value = 6.48%, the device with the CE based solely on the SrRuO_3_ porous deposition = 7.16%, the reference DSSC with standard Pt CE = 7.44%, and the GQD-doped CE allowed DSSC to breach the 8% barrier (8.05%). Similar progression has been recorded by other research teams using CoS [194] or MoS_2_ [195] CEs.

Although optical and electronic properties have attracted the attention of DSSC researchers towards the fabrication of electrodes, it is worth underlining how GQDs could be usefully exploited for the preparation of the electrolyte solution; this is due to the presence of carboxylic groups or amino functions, which provide sufficient stability in an aqueous medium and/or could serve as an anchor moiety to further tune the solubility of these nanomaterials. A very recent article reports a step in this direction, in which Porfarzollah and co-workers report the functionalization of GQDs with ionic liquids, to obtain a quasi-solid-state hybrid electrolyte for DSSC technology [196]. J_SC_ values for the cells using ionic liquid-functionalized GQDs guaranteed superior performance (up to 19.57 mA cm^−2^) if compared with devices based on either pristine GQDs or ionic liquids. Such performance is due to the inhibition of the back-electron transfer phenomena to the electrolyte, which increased the electrons’ lifetime and reduced the recombination reaction rate. Such a design is worth exploiting in the future considering that the limitations of designs based on volatile liquid electrolytes could be easily overcome in this way, providing devices of superior long-term stability and higher photoelectric conversion efficiency.

### 4.2. GQDs as Active Components in Energy Storage Devices

The development of DSSCs as well as other solar cell designs cannot proceed regardless of the development of buffer devices, able to supply energy to a system for a certain time, when the primary power supply is unable to provide electricity. Supercapacitors (SCs) are an intensely investigated technology due to the elevated power capability and large energy density that they can store compared to traditional capacitors [197]. Considering their simplest design [198,199], supercapacitors can be described as being composed of a couple of electrodes, where the space in between is occupied by an electrolyte solution, with an ion-permeable membrane separating the liquid phase into two halves. When connected to an external power source, SCs store charge due to the formation of electrical double layers at the electrode surface (where ions from the electrolyte solution contribute to the formation of the layers), and electrochemical pseudocapacitance. From this brief description, it is immediately evident that the electric properties of the electrodes’ materials are a fundamental issue in this research field, concurrently with the large surface area (wider double layers and higher double-layer capacitance) and chemical stability (electrodes must be inert along with the device life cycle). Several scientists have recognized how GQDs can fulfill these requirements, which, to some extent, recall the criteria followed for the development of GQD electrodes in DSSC investigations (see the previous subsection). Some investigations pointed out how the application of GQDs in supercapacitors could benefit from the accurate synthetic design, as small GQDs can work at a high scan rate and with an ultrafast power response (in the example by Liu et al. [200], GQDs with mean size = 4.8 nm returned a power response = 63.3 μs).

In 2015, the research developed by Mondal and others showed the further potential of GQDs when combined with materials to obtain innovative composites for electrodes [201]. Poly(aniline) was doped with GQDs with average size = 6 nm, and the fibrous aggregate showed during galvanostatic charge–discharge (GCD) measurement a capacitance value of ~1045 F g^−1^ at J = 1 A g^−1^ as well as 80.1% performance after 3000 cycles. A more unique and complex design ensured better performance, with Wang et al. combining NiCo_2_S_4_ with tryptophan-decorated GQDs [202]; in their work, the hybrid material managed to improve the specific capacitance up to ~1450 F g^−1^ at J = 20 A g^−1^, reaching a elevated rate capability (~460 F g^−1^ at J = 1 A g^−1^) and considerable energy density (67.9 W h kg^−1^ at the power density of 800 W kg^−1^), confirming the advantageous use of GQDs in supercapacitor technology. Similarly, Luo and others prepared electrodes relying on transition metal chalcogenides (TMCs); this study is based on the combination of tremella-like NiCo_2_O_4_ with GQDs, with an average size between 2 and 4.5 nm [203]. Brunauer–Emmett–Teller (BET) measurements revealed that the so-prepared material specific surface area was 149 m^2^ g^−1^, and it exhibited excellent specific capacitance (1242 F g^−1^ at 30 A g^−1^ in 2.0 M KOH electrolytes) as well as outstanding capacitance retention (up to 99% after 4000 cycles). In another example, Zhang and collaborators combined MnCo_2_O_4.5_ with GQDs, but, in this case, the attention was directed to the best nanoform for the TMCs to interact with GQDs [204]. A nanoneedle-like structured electrode with absorbed dots was revealed to deliver the largest capacitance (1625 F g^−1^ at 1 A g^−1^), which was four-times higher than the capacitance of a pure MnCo_2_O_4.5_. Looking for cheaper materials, Jia and others reported on less unique hybrids, composed of GQDs and MnO_2_ nanosheets [205]: the specific capacitance is not as high as the previous example (1170 F g^−1^ at a scan rate of 5 mV s^−1^), but the stability that they observed for their materials is notable, with 92.7% performance retained after 10 000 cycles under 0–1.3 V scans. Moreover, a prototype asymmetric SC was assembled according to the GQDs/MnO_2_-3//nitrogen-doped graphene design, which showed elevated energy density (118 W h kg^−1^ at power density = 923 W kg^−1^). Other nanostructured MnO_2_ forms were investigated as well, with electrodes based on the combination of GQDs and needle-like MnO_2_ [206] returning electrodes with 2712 mF cm^−2^ at a current density of 1.0 mA cm^−2^. In another study [207], Zheng et al. surpassed the 2000 specific capacitance threshold as well, using Ni(OH)_2_ nanosheets mixed with amino-functionalized GQDs (2653 F g^−1^ at 1 A g^−1^).

From an environmentally friendly and sustainable technological development perspective, it is worth considering naturally available substrates, even for these cutting-edge applications. GQDs have been reported to decorate halloysite tubes [208], with acceptable overall performance (GQD-HNT 6 A/g 5000 88% performance retention after 5000 cycles at J = 6 A g^−1^). Qiu et al. prepared a composite composed of histidine-functionalized GQDs and Ni-Co-layered double hydroxides, this time serving as a positive electrode in a prototype supercapacitor [209]; in this case, the device showed the maximum energy of 48.89 W h kg^−1^ at 0.80 kW kg^−1^, as well as interesting cycling stability (91.13% after 6000 cycles), confirming similar results obtained by Ma and others [210]. Flexible fiber-type solid-state supercapacitors have been recently presented for wearable soft electronics based on carbon fiber, which is used as a tridimensional support and as a source of GQDs after degradation in the presence of HNO_3_/H_2_SO_4_ [211]. The so-produced composite demonstrated high performance and high flexibility as well, with performance retention over 1000 bending cycles at 180°, up to 99.5% capacity.

**Figure 8:**
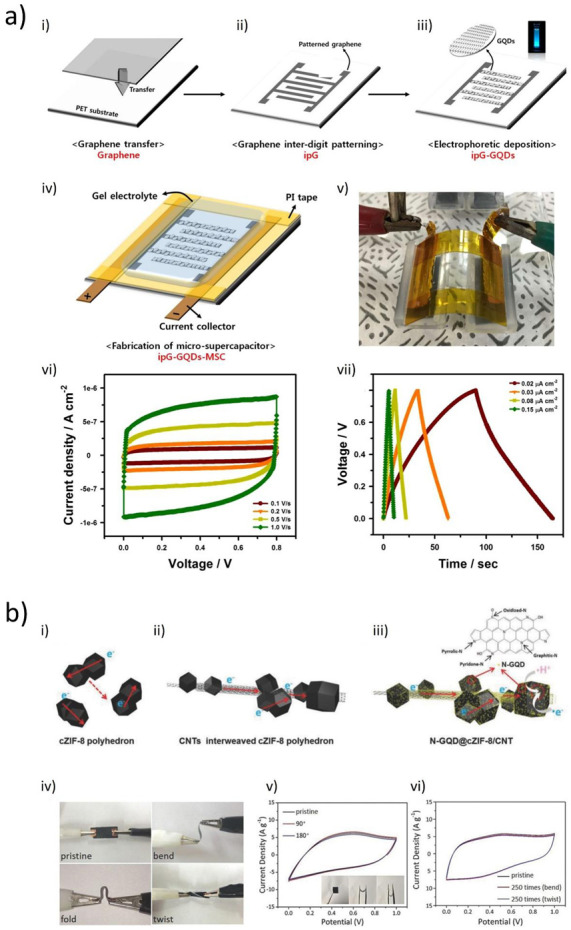
Supercapacitor architectures integrating GQD-based electrodes. (**a**) Schematic illustration for fabrication of an ipG-GQD-MSC: (**i**) Monolayer graphene prepared by CVD is transferred onto a PET substrate; (**ii**) the graphene is patterned to prepare interdigitated graphene electrodes, ipG; (**iii**) GQDs are deposited onto the ipG by using an EPD method: ipG is immersed in a GQD dispersion including Mg(NO_3_)_2_ and a DC voltage is applied, yielding ipG-GQDs; (**iv**) illustrations of a fabricated ipG-GQD-MSC; (**v**) optical image of an ipG-GQD-MSC under bending; (**vi**) cyclic voltammetry curves collected under various scan rates; (**vii**) galvanostatic charge–discharge curves collected under various current densities. Figure adapted from Reference [213]. Copyright 2016, Elsevier B.V. (**b**) Schematic diagram of (**i**) the rational design, (**ii**) the electronic transport, (**iii**) the proton-coupled electron transfer mechanism of N-GQD@cZIF-8/CNT composite, (**iv**) the flexible device based on N-GQD@cZIF-8/CNT under different bending conditions and (**v**-**vi**) the corresponding CV curves measured for the device in different conditions at a scan rate of 50 mV s^−1^. Figures adapted from Reference [218]. Copyright 2018, John Wiley & Sons, Inc.

Looking at reports where the focus is mainly shifted to the fabrication of a prototype device, an interesting work was published in 2015 by Shen and collaborators, where an all-solid-state microsupercapacitor was developed [212]. The study is interesting from several perspectives, such as the miniaturization of the device, the use of ionic liquids to bypass any failure due to the volatile nature of the electrolyte solution solvent, and also for the materials of choice; here, GQDs were used in the negative electrode due to their chemical inertia and large surface area (the design is asymmetrical and MnO_2_ nanosheets are placed at the opposite electrode), and the performance was evaluated using a range of ionic liquids. Great attention was given to the choice of ionic liquid, with the best-performing device using 1-ethyl-3-methylimidazolium bis(trifluoromethylsulfonyl)imide ([EMIM][NTF2]) with 4 wt.% fumed SiO_2_. Excellent rate capability with a scan rate of up to 2000 V s^−1^ were recorded, as well as an ultrafast frequency response (τ_0_ = 206.9 μs) and high energy density. In contrast, Lee and coworkers looked at the possibility of developing highly transparent and flexible micro-supercapacitors based on interdigitated graphene patterns deposited with GQDs by means of electrophoretic deposition [213]. Their approach allowed them to fabricate devices characterized by high transparency (~93% at 550 nm), suggesting potentially simple integration in the automotive sector as well as in window technology; furthermore, the specific capacitance was 9.09 μF cm^−2^ and they kept their capacity unaltered after 10,000 charge/discharge cycles. High stability was revealed under severe bending angles (45°) after 10,000 cycles (87.9% retained capacity; see Figure 8a).

In the previous discussion, the focus was on “standard GQDs” because there are few reports exploiting the potential of doped-GQDs; however, since 2016, it has been known that supercapacitors could benefit from better performance due to the trap states created by both dopants and edge states, which can adsorb charge carriers to enhance the storage capacity [214]. In 2017, Li et al. documented the construction of SC electrodes based on a conductive, flexible, free-standing, 3D carbon nanotube/carbon cloth network, where highly N,O co-doped GQDs were electro-deposited [215]. The electrode showed a specific capacitance of 164.9 F g^−1^ at a current density of 0.5 mA cm^−2^, and the SC based on this electrode was shown to maintain a capacity of 87.5% after 2000 charge/discharge cycles; further optimization and miniaturization allowed the authors to fabricate micro-supercapacitors retaining 82.4% of their initial capacity after 5000 cycles [216]. The same authors explored the possibility of coupling N-GQDs with other scaffolds to achieve better performance, such as carbonized metal–organic frameworks cMOF-5 [217] and cZIF-8 [218], the latter being the best option, with the final electrodes (N-GQDs@cZIF-8/CNT) having a specific capacitance of 540 F g^−1^ at 0.5 A g^−1^ in a 1 M H_2_SO_4_ electrolyte and excellent cycle stability (90.9% over 8000 cycles; see Figure 8b). Moreover, 3D ordered reduced graphene oxide was recently explored as a scaffold for N-GQDs in electrode fabrication [219], underlining the vitality of this research field.

## 5. Conclusions and Perspectives

The increasing demand for innovative materials for new technological applications has forced the scientific community to devote huge efforts to the investigation of new strategies. GQDs are one of the most relevant results of 20 years of research—a material in which excellent optical and mechanical features, chemical inertia, and satisfying processability are combined in a single object. To properly modulate such features, a broad variety of synthetic designs have been established, returning a number of protocols that allow fine-tuning of GQDs’ size, energy bandgap, light absorption, and emission profiles, making the integration of GQDs into several applications possible and profitable. Several approaches have been investigated, ranging from classical chemical synthesis to innovative strategies such as microwave irradiation or sonochemistry (Section 2.2.5), to make dots synthesis more sustainable from an environmental perspective. Moreover, considering the promising N-doped GQDs, production approaches benefit from eco-friendly strategies avoiding the use of hazardous and toxic chemicals, addressing the concern of the global scientific community for the health of the planet.

Regarding the applications, GQDs have recently found broader application in cutting-edge technologies, and research areas related to the energy sector have given notable value to these nanosized carbonaceous materials, due to the possibility of fabricating efficient and low-cost devices exploiting natural raw materials as GQD sources, as was underlined in the review; moreover, they can be easily integrated with other established materials in order to isolate composites of astonishing performance. Moreover, compared with other carbon nanoforms, the controlled synthesis of GQDs is a powerful tool, able to offer strict control over their structural features, size, and optical profile, thus providing a material that is much more homogeneous with respect to these characteristics, a condition that is desired in high-tech fields and is far from being met by other carbon nanomaterials.

Although this single review cannot cover every possible aspect of interest within specific GQD research areas, we hope that we have offered a comprehensive and valuable overview of the synthetic strategies that are currently under development, with a rational analysis of the most recent literature in the field. The rapid progression in the field within the scientific community necessitates a constant update of the state-of-the-art both in terms of chemical strategies and achievements in the exploitation of GQDs in application fields, but we hope that our presentation offers a solid basis for future reports.

## Figures and Tables

**Table 1:** Precursors and properties of GQDs produced using bottom-up methods.

Samples	Precursors	Reaction Conditions	Reaction Yield (wt.%)	Size Distribution	Average Diameter (nm)	PL QY (%)	Color	I_D_/I_G_ Ratio
GQDs [36]	Glucose	Autoclave, 200 °C, 8 h	-	Uniform dispersion	8	-	Green, 540 nm	/
GQDs [40]	Humic acid	Autoclave, 180 °C, 5 h	-	Monodispersed spheres	4	5.2	Blue, 457 nm	0.83
GQDs [38]	CA	200 °C, 35 min	-	/	15	9	-	/
GQDs [35]	Acetylacetone	200 °C, 800 W, 5 min 200 °C, 900 W, 5 min	- -	/ /	5 2.3	1 3.4 *	Green Blue	1.08
GQDs [50]	Poly(acrylonitrile)	100 °C, 24 h, H_2_SO_4_/HNO_3_, ultrasonic peeling 40 kHz, 2 h, stirring 100 °C, 24 h,	-	/	10.98	-	Blue	0.41
N-GQDs [54]	CA + EDA	Autoclave, 140–190 °C, 5–8 h	-	/	5–10	75.2	Blue	/
K-doped GQDs [87]	Sucrose + KOH	Autoclave, 170 °C, 4 h	-	/	3–4	-	544 nm	/
B-doped GQDs [85]	TNP + borax	Autoclave, 120–200 °C, 2–10 h	71	/	2	16.8 *	Green	/
Cl-GQDs [88]	Fructose + HCl	170 °C, 4 h	-	Monodispersed	5.4	6.8	Blue to white, orange, green, and red	/
S,P-doped GQDs [84]	Sodium phytate + Na_2_SO_4_	180 °C, 7 h	-	/	3.5	15.7	440 nm	/
S,N-doped GQDs	CA + TU [74,75,76] P3AT [77] CA + U [78] TNP + TU [79] CA + L-cysteine [80]	160 °C, 4 h 170 °C, 20 h 200 °C, 10 h Oil bath, 90 °C, 6 h Autoclave, 180 °C, 6 h	- - 43.2 [78] - -	Narrow size distribution	3.1–10 / / / /	22.2–71 / / / /	Blue, green, and red / / / /	0.77–0.94 / 0.99–1.02 / /
S/N co-doped GQDs [81]	CA + TU + (NH_4_)_2_SO_4_	IR furnace (80 kW m^−2^), 280 °C for 30 min	41	4–10	6.2	25.5	Blue	0.85
N,S-GQDs [83]	CA + TU	Hydrothermal, 180 °C, 6 h	/	Narrow size distribution	3.10 ± 0.54	/	/	/
S-GQDs [89]	Durian	Hydrothermal, 150 °C, 12 h	15	/	2–6	/	Orange	0.32
Bf-GQDs [90]	Bamboo cellulose nanocrystals	Hydrothermal, 180 °C, 8 h	57	/	9.8	38.9	410 nm	0.78
LGQDs [91]	Alkali lignin	Alkali lignin + *o*-ABSA, 80 °C, 20 min	/	/	2.20 ± 0.40	20.7	380 nm	0.98
M-GQDs [92]	Miscanthus	Hydrothermal 200 °C, 12 h	19.8	Uniform dispersion	4.05 ± 0.61	20.2	Blue	1.09

* absolute QY.

**Table 2:** Summary of top-down synthesis methods and properties of GQDs.

Samples	Method	Starting Material	Average Diameter (nm)	Quantum Yield (%)	Production Yield (wt.%)	Color
GQDs [101]	Chemical oxidation	C_60_	7–10	-	-	Yellow–red
GQDs [109]	Chemical oxidation	C_60_	1–2.5	-	-	-
GQDs [112]	Chemical oxidation	Graphene oxide	2.5	-	-	-
GO-QDs [111]	Chemical oxidation	Aphanitic graphite	4.5	-	40	Orange
GQDs [113]	Hydrothermal	GO	~4	-	-	Violet–green
FeGQDs CoGQDs Ni GQDs [114]	Hydrothermal	Graphene capsules	15–25	11.7 12.4 12.2	14–17 - -	Yellowish - -
N-GQDs [115]	Hydrothermal	Graphene oxide	1.84 ± 0.28	46	20	Blue
F-GQDs B-GQDs G-GQDs Y-GQDs O-GQDs R-GQDs [117]	Hydrothermal	Graphene oxide	~3.7 ~2.7 ~3.4 ~4.1 ~4.5 ~5.1	12.9 18.3 41.4 38.3 37.5 18.7	60 - - - - -	Blue Blue Green Yellow Orange Red
GQDs [119]	Solvothermal	Expanded graphite	35	15	-	Blue
Edge N/O-GQDs [121]	Solvothermal	Graphite	6–16	19.1	-	Sky blue
NGQD-d NGQD-w [122]	Solvothermal	Graphite flakes	5	63.8 26.2	46 6	Green Blue
GQDs [127]	Electrochemical oxidation	Graphene oxide	2.4 ± 0.3, 3.6 ± 0.2, 4.6 ± 0.4	7.8	65.5	Purple–blue Blue Green
N-GQDs [128]	Electrochemical oxidation	N-CNT/N-graphene hybrid	3.6 ± 1.3	19.3	82.1	Blue
RF-GQDs [129]	Electrochemical oxidation	Graphite	3	-	-	Red
P-GQDs [130]	Electrochemical oxidation	Graphite rods	2–4	-	-	-
GOQD GQD [131]	Electrochemical oxidation	High-defect graphite rods	2.24–3.04 -	- -	- -	Green, blue–green Blue–green, blue
GQDs_(3V)_ GQDs_(5V)_ GQDs_(7V)_ [134]	Electrochemical oxidation	Graphite electrode	2.9–5.2	9.5 11.2 4.6	- - -	Blue
p-GQDs [139]	Electrochemical oxidation	Graphite electrode	24.0	2.07	-	Purple–blue
gGQDs bGQDs [141]	Microwave- assisted chemical oxidation	Graphene oxide	4.5	-	-	Green Blue
GQDs [142]	Microwave- assisted hydrothermal reduction	Graphene oxide	2–8	-	-	Blue
F-GQDs [143]	Microwave- assisted chemical oxidation	F-GO	5.6	7.5	-	Bright blue
GQDs [33]	Ultrasonic-assisted oxidation	Graphene oxide	4–10	-	-	-
pGQDs [144]	Ultrasonic/ supercritical CO_2_/ water exfoliation	Pristine graphite	2–4	-	50	UV blue
LA-GQDs [145]	Liquid-phase laser ablation	Carbon nano-onions	4.1(8)	-	-	Blue
GQDs N-GQDs_(T65)_ N-GQDs_(T90)_ N-GQDs_(T120)_ [150]	Liquid-phase laser ablation/ solvothermal	Cryomilled graphite	4.5	0.60 0.91 1.74 4.05	- - - -	- - - -

## Data Availability

Not applicable.
